# Detection and management of postpartum haemorrhage: Qualitative evidence on healthcare providers' knowledge and practices in Kenya, Nigeria, and South Africa

**DOI:** 10.3389/fgwh.2022.1020163

**Published:** 2022-11-18

**Authors:** Shahinoor Akter, Gillian Forbes, Suellen Miller, Hadiza Galadanci, Zahida Qureshi, Sue Fawcus, G. Justus Hofmeyr, Neil Moran, Mandisa Singata-Madliki, Taiwo Gboluwaga Amole, George Gwako, Alfred Osoti, Eleanor Thomas, Ioannis Gallos, Kristie-Marie Mammoliti, Arri Coomarasamy, Fernando Althabe, Fabiana Lorencatto, Meghan A. Bohren

**Affiliations:** ^1^ Gender and Women’s Health Unit, Centre for Health Equity, University of Melbourne School of Population and Global Health, Carlton, VIC, Australia; ^2^ Centre for Behaviour Change, University College London, London, United Kingdom; ^3^ Department of Obstetrics, and Reproductive Sciences, School of Medicine, University of California, San Francisco, United States; ^4^ Africa Center of Excellence for Population Health and Policy, Bayero University Kano, Kano, Nigeria; ^5^ Department of Obstetrics and Gynaecology, University of Nairobi, Nairobi, Kenya; ^6^ Department of Obstetrics and Gynaecology, University Cape Town, Cape Town, South Africa; ^7^ Department of Obstetrics and Gynaecology, University of Botswana, Gaborone, Botswana; ^8^ Effective Care Research Unit, Department of Obstetrics and Gynaecology, Universities of Witwatersrand and Walter Sisulu University, East London, South Africa; ^9^ KwaZulu-Natal Department of Health, and Department of Obstetrics and Gynaecology, School of Clinical Medicine, College of Health Sciences, University of KwaZulu-Natal, Durban, South Africa; ^10^ Africa Center of Excellence for Population Health and Policy, Bayero University Kano and Department of Community Medicine, Bayero University/Aminu Kano Teaching Hospital, Kano, Nigeria; ^11^ Department of Global Health University of Washington, United States; ^12^ WHO Collaborating Centre on Global Women’s Health, Institute of Metabolism and Systems Research, College of Medical and Dental Sciences, University of Birmingham, London, United Kingdom; ^13^ UNDP/UNFPA/UNICEF/wHO/World Bank Special Programme of Research, Development and Research Training in Human Reproduction (HRP), Department of Sexual and Reproductive Health and Research, World Health Organization, Geneva, Switzerland

**Keywords:** maternal health, postpartum haemorrhage, maternal mortality, qualitative research, formative research, clinical care bundles

## Abstract

**Background:**

Postpartum haemorrhage (PPH) is the leading cause of maternal death globally. Most PPH deaths can be avoided with timely detection and management; however, critical challenges persist. A multi-country cluster-randomised trial (E-MOTIVE) will introduce a clinical care bundle for early detection and first-response PPH management in hospital settings. This formative qualitative study aimed to explore healthcare providers' knowledge and practices of PPH detection and management after vaginal birth, to inform design and implementation of E-MOTIVE.

**Methods:**

Between July 2020–June 2021, semi-structured qualitative interviews were conducted with 45 maternity healthcare providers (midwives, nurses, doctors, managers) of nine hospitals in Kenya, Nigeria, and South Africa. A thematic analysis approach was used.

**Results:**

Four key themes were identified, which varied across contexts: in-service training on emergency obstetric care; limited knowledge about PPH; current approaches to PPH detection; and current PPH management and associated challenges. PPH was recognised as an emergency but understanding of PPH varied. Early PPH detection was limited by the subjective nature of visual estimation of blood loss. Lack of expertise on PPH detection and using visual estimation can result in delays in initiation of PPH management. Shortages of trained staff and essential resources, and late inter-hospital referrals were common barriers to PPH management.

**Conclusion:**

There are critical needs to address context-specific barriers to early and timely detection and management of PPH in hospital settings. These findings will be used to develop evidence-informed implementation strategies, such as improved in-service training, and objective measurement of blood loss, which are key components of the E-MOTIVE trial (**Trial registration:** ClinicalTrials.gov: NCT04341662).

## Introduction

Postpartum haemorrhage (PPH) is one of the leading causes of severe maternal morbidity, and the leading cause of direct maternal deaths, accounting for 27% of global maternal deaths (1–3). In 2017, PPH was responsible for the deaths of 127,000 women worldwide (2), despite being largely a preventable and treatable condition (4). PPH can occur in any setting; however, PPH-associated mortality is disproportionately higher in low and middle-income countries (LMICs) compared to high-income countries (5–7). A World Health Organization (WHO) systematic analysis of causes of maternal deaths, revealed that nearly 73% of maternal deaths in LMICs are caused by PPH, and over 40% of PPH deaths occurred in sub-Saharan Africa alone (8). When PPH occurs, early recognition and timely, evidence-based management are crucial to saving women's lives (8, 9).

The most widely accepted definition of PPH is based on the amount of blood lost after birth (10). For a vaginal birth, PPH is defined as blood loss of ≥500 ml and severe PPH is defined as loss of ≥1,000 ml (10). Primary PPH, the most common form of PPH, occurs within 24 h following childbirth; secondary PPH occurs between 24 h and 6 weeks after birth (11, 12). While some women have identifiable risk factors for PPH, such as a history of PPH, approximately 20% of PPH cases occur in women without risk factors (11). Therefore, healthcare providers must have the knowledge, skills, and resources to detect and manage PPH for each birth (11, 12). However, healthcare providers may miss opportunities to detect PPH early, delaying the use of effective interventions for PPH management leading to avoidable maternal deaths (3, 4, 13).

Visual estimation of blood loss is the most common method for PPH detection following vaginal birth (14); however, it is inherently subjective and unreliable as both over- and under-estimation are reported (9, 15). Under-estimation is reported more frequently, with higher volumes of blood lost than healthcare providers' estimate, thus delaying PPH detection and management (9, 16). Moreover, early detection requires regular monitoring of the woman after birth, which can be challenging for healthcare providers in low-resource settings where essential resources for PPH care are limited, maternity wards are frequently understaffed, and patient volumes are high (17). Limited awareness or access to current PPH treatment guidelines, limited essential resources for PPH care, limited opportunities for refresher training (18, 19), and understaffing can lead to inconsistent and delayed use of evidence-based, effective first-line PPH interventions (uterotonics, tranexamic acid, intravenous fluids, and uterine massage) (3, 4, 10, 13).

### The E-MOTIVE research programme

The E-MOTIVE research programme is a collaboration between 17 universities and non-governmental organisations across ten countries. The programme aims to improve PPH detection and first response management for PPH, through the implementation of a new clinical care bundle called “E-MOTIVE” (20). E-MOTIVE was designed to use by healthcare providers for women with PPH following vaginal birth. In this paper the term “PPH” will be used to refer to PPH following vaginal birth only, as PPH following caesarean birth is outside the context of the present study. The E-MOTIVE bundle was based on a WHO technical consultation (3) using the globally recognized and utilized WHO Guidelines for Prevention and Management of PPH (10, 21, 22). The E-MOTIVE care bundle is composed of six evidence-based components: (1) ***E***arly detection of postpartum haemorrhage using an under-buttock, calibrated blood collection drape; (2) ***M***assage of the uterus; (3) administration of ***O***xytocic drugs, (4) administration of ***T***ranexamic acid (TXA); (5) administration of ***I***ntra***V***enous fluids, and (6) **E**xamine genital tract, placenta, lab work, and bladder with ***E**scalation* when necessary if the primary response bundle does not stop bleeding (20). The interventions in the E-MOTIVE care bundle are designed to be administered concurrently or in rapid succession to every woman presenting with PPH. The E-MOTIVE bundle also includes “implementation strategies” to improve teamwork, communication, and cooperation (20). The E-MOTIVE project will take place in three key phases: (1) formative phase, (2) parallel cluster randomised trial with baseline control (trial registration: NCT04341662), process evaluation, and cost-effectiveness assessment, and (3) post-intervention phase to update clinical guidelines.

Implementing new care bundles in clinical practice can be complex, and likely requires strategies to support implementation – beyond simply disseminating the bundle (3). To decide how to best support implementation, it is critical to first understand current practice, including attitudes and perceptions towards PPH and the bundle (3, 23). The formative phase of E-MOTIVE aims to do this by using a mixed-methods approach to better understand maternity care providers' current clinical practices of PPH detection and management. This paper presents an analysis of the formative qualitative data from Kenya, Nigeria, and South Africa (20), three countries with a high burden of PPH and PPH-related maternal mortality (Table 1) (20). These three countries represent different geo-political contexts, cultures, and variations in maternal health outcomes (24), which provides different insights regarding PPH detection and management among healthcare providers and will contribute to the development of more effective implementation strategies to support behaviour change and uptake of the bundle. In this paper and the E-MOTIVE Research Programme, we focus on detection and first response management to PPH.

**Table 1:** Characteristics of the study countries: trends in the maternal mortality ratio (MMR) and proportion of maternal deaths due to PPH.

Country	Recent trends in MMR^a^	Maternal deaths during postpartum period or due to PPH
Kenya	MMR decreased between 2000 and 2017 from 708 to 342 per 100,000 live births (2017 UI: 253 to 476) (6).	Obstetric haemorrhage was found to be the underlying cause of 192, or 49% of the 945 maternal deaths during this period (25)
Nigeria	Fourth highest MMR globally, estimated number of maternal deaths in 2017, with nearly 67,000 maternal deaths and accounted for 23% of global maternal death (917; UI 658 to 1320) (6).	PPH was reported as 2.2% of births over a one-year period in 42 tertiary hospitals and was the most frequent obstetric complication across all hospitals. Nearly 42% of maternal deaths resulted from PPH (26)
South Africa	MMR decreased between 2000 and 2017 from 160 to 119 per 100,000 live births (2017 UI: 69 to 153) (6).	PPH identified as the 3rd leading cause of maternal deaths between 2014 and 2017, accounted for 16.9% (*n* = 624) of the total maternal deaths during this period (27)

^a^ Maternal mortality ratio; UI, uncertainty interval.

## Methods

### Study design

The formative study protocol has been published previously (20). We used a qualitative descriptive approach using in-depth interviews, to discover and understand the perspectives of the people involved by describing their experiences about PPH care (28, 29). We report this study based on the Standards for Reporting Qualitative Research (30).

### Study sites

The qualitative study was conducted in a convenience sample of three hospitals per country (*n* = 9) in Kenya, Nigeria, and South Africa. Table 1 presents an overview of the study countries' recent trends in overall maternal mortality ratios and PPH-related maternal mortality. We selected hospitals using maximum variation sampling to ensure variation in location, and burden of PPH (20).

### Study participants

We aimed to explore perspectives of maternity healthcare providers from different cadres, including midwives, nurses, junior doctors, medical officers, trainees, and obstetricians. Hospital administrators and managers in charge of the maternity wards (such as head of obstetrics and matron-in-charge) were also included if they were trained clinicians. All participants could speak English.

### Sampling and recruitment

Participants were recruited using a maximum variation, purposive sampling strategy to achieve a stratified sample without random selection and to ensure heterogeneity and diversity (31). In line with qualitative sample size guidelines based for thematic analysis 45 qualitative interviews were conducted with healthcare provider from different cadres: 15 per country with 5 per hospital. After 45 interviews, it deemed to have sufficient data about PPH detection and management (32, 33). Given that the study took place during the COVID-19 pandemic, the country investigators and research teams facilitated contact with the healthcare providers and administrators at the study hospitals in person, or *via* email, telephone, or video conferencing software. Each participant was given a plain language information sheet, invited to participate by the research team, and, if they agreed, provided written informed consent.

### Data collection procedures

Data were collected between July 2020 and June 2021 (Nigeria: July to September 2020, Kenya: October to December 2020, South Africa: May to June 2021). Qualitative interviews were conducted either face-to-face (where possible and safe depending on COVID-19 restrictions) or virtually (using Zoom or telephone). Interview time, location, and mode of interview (virtual or face-to-face) were decided by the research team and participants based on their preferences and existing COVID-19 restrictions. Face-to-face interviews lasted 45–60 min, and virtual interviews lasted 60–90 min (some virtual interviews had poor connectivity which resulted in the interviews taking longer). Qualified and trained healthcare professionals (midwives and doctors) and social scientists from each of the country teams conducted all interviews. A two-day training workshop for each country team was conducted to familiarize them with the project and interview topic guide. The topic guide was pretested and modified (where needed) through discussion and consensus among the research teams, including country teams, before starting data collection. All interviews were conducted in English and audio-recorded with permission.

Our research team was multidisciplinary, from diverse, international academic and professional backgrounds with a range of research focus areas and expertise that included medical anthropology, social and behavioural sciences, midwifery, nursing, public health, and medicine. Each country's research team had members with clinical and/or social science backgrounds and were involved throughout data collection and analysis. Interviews were conducted and transcribed verbatim by qualified and trained members of each country's research team, typically the same person who conducted the interview. Interviewers' reflexive notes complemented the analysis by helping to contextualize what happened during the interview. Given that all interviewers were local and predominantly health professionals, there was a chance that their personal and professional experiences about the topic of interest might impact their impartiality as interviewers (34, 35). Reflexive journaling was used by the interviewers to reflect and record their thoughts and feelings and thus bracket their perceptions and subjectivity. This reflexive practice was continued throughout data collection and analysis, which allowed the researchers from the formative team to be conscious of their personal experiences and perspectives, and to portray the beliefs and experiences of the participants more accurately. Interviewers were also actively involved throughout the analysis to ensure the appropriateness of the interpretations.

### Study instrument

The interview guide (see Supplementary Appendix 2) was structured according to three broad sections: (1) how is PPH currently detected and managed for a vaginal birth, (2) factors influencing current practice for PPH management, and (3) factors potentially influencing the implementation of the E-MOTIVE bundle. We used two related behaviour change and implementation science frameworks [Capability, Opportunity, Motivation and Behaviour (COM-B) (23) and theoretical domains framework (TDF)] (36) to design the semi-structured interview guides, in order to structure lines of enquiry barriers and enablers to changing clinical practice behaviours (20). In this paper, descriptive findings related to PPH detection and management will be reported, and findings related to factors influencing the implementation of the E-MOTIVE care bundle will be reported elsewhere (37). Using the semi-structured topic guide assisted interviewers in gaining a comprehensive understanding of the topics and helped to ensure a common line of inquiry across interviews for analytical comparisons. The flexible nature of the guide also permitted discussion to be conversational where participants could answer the questions at length and relevant prompts were used to elaborate ambiguous responses (38), and also ensured data richness.

### Data analysis

Thematic data analysis was used following a stepwise method developed by Braun and Clarke using a range of strategies to enhance rigor (33, 39). The interviews were read through several times separately by SA and GF, who then double coded five interviews. Transcripts were coded for words and phrases relevant to the research questions, and data were collated to each code. We started analysing the Nigeria data, and used codes developed from the Nigeria data to inform the analysis of data from South Africa and Kenya, adding new codes where necessary. Frequent debriefing sessions involving all co-authors were held to test the ideas and interpretations and recognise individual biases and preferences. As both SA and GF are social and behavioural scientists, a member of the research team with a clinical background (SM) reviewed all clinical data related to PPH detection and management during analysis. Furthermore, the initial code list with identifying themes and subthemes were also shared and discussed with the co-author team to check if final categories and definitions were aligned with and reflected what was shared during interviews. After coding was completed, the formative research team compared findings across sites and countries. Throughout the analysis process, the research team met weekly to negotiate any disagreements and to clarify and refine the categories ensuring a coherent pattern. After conducting 15 interviews in each country (total 45 in three countries), data sufficiency was deemed to have been achieved as no new themes or sub-themes were reported in the last 3 interviews from each country (33).

## Findings

A total of 45 participants are included in this analysis (Table 2), comprised of 17 doctors (general medical doctors; obstetrician-gynaecologists; interns), 6 nurses, 14 midwives or nurse-midwives, and 8 administrators (all with clinical backgrounds). Across professions, almost all midwives and nurses (*n* = 18/20) and about half of doctors (*n* = 8/17) were women. All participants worked in the maternity wards in the study hospitals and had a wide range of years of work experience.

**Table 2:** Characteristics of healthcare providers (*N* = 45) who participated in the qualitative study from Kenya, Nigeria and South Africa.

Participants details	Kenya	Nigeria	South Africa	Total
	*n* = 15	*n* = 15	*n* = 15	*n* = 45
**Professions**				
Doctors^a^	6	6	5	17
Nurses	1	4	1	6
Midwives^b^	6	2	6	14
Administrative^c^	2	3	3	8
**Gender**				
Male	7	5	7	19
Female	8	10	8	26
**Professional years of experience**	Range 2-13 years	Range 1-30 years	Range 0.5-20 years	

^a^ Doctors include general medical doctors, interns, obstetricians/ gynaecologists.

^b^ Midwives include midwives, nurse-midwives, advanced midwives.

^c^ All administrative participants had medical background Administrators include in-charge, Head of school, in-patient coordinator).

**Figure 1:**
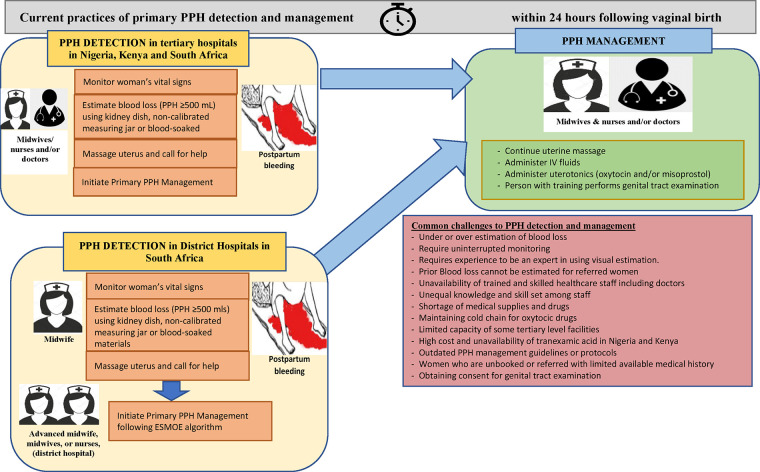
Current practices and key challenges about detection and primary PPH response in nine hospitals in Kenya, Nigeria, and South Africa, based on the findings from 45 interviews.

Using an inductive thematic analysis approach (39), four distinct but related themes were identified including (1) in-service training on PPH or emergency obstetric care, (2) defining and detecting PPH, (3) current practices of PPH detection and associated challenges, and (4) primary management of PPH and associated challenges. Findings that are consistently reported by different cadres of healthcare providers (doctors, nurses, midwives) and across countries are reported together, and results that differ by cadre or country are reported separately. In the following sections, we discuss each of these themes in detail, and Figure 1 depicts current practices and associated challenges of PPH detection and management.

### In-service training on PPH or emergency obstetric care

In clinical education, there is pre-service training (e.g., medical and nursing school), and in-service training. Across the three countries, there was wide variation in in-service training on PPH detection and management, and only participants in South Africa received simulation training on PPH. Most participants across three countries did not receive any in-service training about PPH in the past five years.

Most Nigerian participants had not attended any in-service training on PPH. In Kenya, most participants reported attending national Basic Emergency Obstetric and Newborn Care (BEmONC) in-service training conducted by the Ministry of Health, which included sessions on PPH. Kenyan participants also reported Continuing Medical Education (CME) sessions focusing on reflection, debriefing, lectures and obstetric emergency simulation training (a method for practicing the management of obstetric emergencies including PPH).

*“We hold CMEs … We hold monthly meetings, and this is where we receive feedback in all areas. Including PPH [Nurse, Kenya]*

In South Africa, most participants attended lecture and simulation-based, 3-day ESMOE (Essential Steps in the Management of Obstetric Emergencies, (including PPH) in-service simulation training (40) anywhere from the past year to 7 years ago.

*“It [ESMOE] was mostly the theory in terms of, you know, we went through a lecture covering most obstetric emergencies… and the use of certain drugs. … Then there were also discussions on the statistics in terms of maternal mortality, and, then from a practical point of view… they had instruments there, and we practised how to use them, on models and dolls and stuff.” [Doctor, South Africa]*

Despite in-service training, most participants across three countries expressed interest in receiving additional continuous training on PPH care to be better informed on the latest evidence-based practices. They also emphasised that receiving multidisciplinary simulation training could build effective teamwork by ensuring that all team members would have a similar level of clinical understanding of PPH care and the skills to provide that care. A minority of more experienced participants – mainly those staff with substantial professional experience – believed that they had sufficient training and that efforts should instead be directed to new or lower-cadre staff only.

### Defining and detecting PPH

When asked about their understanding of what constitutes PPH, healthcare providers from different cadres across three countries reported mixed knowledge and definitions (Supplementary Appendix 3). Across all countries, doctors were more likely than midwives or nurses to report definitions of PPH, blood loss thresholds, and vital signs changes that aligned with the criteria in the WHO PPH Guidelines (10, 21, 41).

Most doctors in Kenya and South Africa, who had more years of clinical experience, provided a clinical definition for primary and secondary PPH based on blood loss quantification:

• Primary PPH: ≥500 ml blood loss within 24 h following childbirth, or any amount of blood loss with worrying vital signs;
• Severe primary PPH: ≥1,000 ml of blood loss within 24 h following childbirth;
• Secondary PPH: ≥500 ml blood loss between 24 h and 6 weeks following childbirth;

As well as clinical signs of hemodynamic instability [decreased blood pressure, increased pulse rate, tachypnoea, and altered physical and mental state (level of consciousness)]. In contrast, junior doctors in South Africa reported that they had limited clinical understanding of PPH and limited knowledge in how to detect PPH. In Nigeria, doctors defined different types of PPH based on blood loss, most commonly reporting PPH as a laboratory finding, a packed cell volume (PCV) decrease of >10%. Only one consultant obstetrician in Nigeria reported that a woman's clinical condition was also important to interpret al.ongside blood loss.

*"We are not too rigid as to this [500 ml of blood loss] because the clinical condition of the woman matters a lot. The patient who is anaemic already she mustn't lose up to 500 ml before we classify her as having PPH…Or patient who has sickle cell anaemia. [Consultant Obstetrician, Nigeria]*

A few doctors in Nigeria also reported that PPH may occur in women where there would be no noticeable blood loss (concealed PPH), but their vital signs would be unstable. Most midwives and nurses across the three countries were aware of PPH; however, several offered inconsistent definitions of PPH. For example, most midwives and nurses did not provide any quantification of blood loss instead referring to “*excessive blood following vaginal birth*”. However, all mentioned changes of signs and symptoms of hemodynamic instability and shock (unstable blood pressure, pulse rate, pallor, dizziness).

### Current practices of PPH detection

Healthcare providers across three countries shared their knowledge and practices about PPH detection, including knowledge of risk factors for PPH, steps taken to detect PPH, different staff roles in detection, the role of visual estimation of blood loss, the associated challenges of using visual estimation, and overall challenges of PPH detection. Participants noted that they often anticipated which women might have PPH based on their medical history, obstetric history, or complications arising during the current labour and birth. Risk factors mentioned included a previous history of PPH, large baby, multigravida, placental problems, or a history of coagulopathy. Conversely, participants stated that detecting PPH and assessing blood loss or causes of blood loss were more complicated when women arrived unbooked or were referred in with no health records and experiencing severe PPH. The participants would not know how much blood had already been lost, how much time since the bleeding began, or if the woman had co-morbidities or other risk factors, thus complicating PPH management.

*"There are patients who come in without any antenatal profile and are almost in the second stage of labor. They come, deliver [the baby] and go back to bed then shortly you hear the mother has fainted … Since the mother did not have an antenatal profile or if they had their HB was initially low and so even if they lost a little blood but because their volume is low, so it affects them more, so those are the difficult situations to detect PPH.” [Nurse, Kenya]*

The steps to detect PPH for women who laboured and gave birth in the hospital varied slightly by country. In Nigeria and Kenya, the person who supported the woman during labour and childbirth (mainly midwives, occasionally doctors), would identify PPH in the delivery ward. Occasionally due to high volumes of women and low staffing in Nigeria, women or family members might need to inform midwives of heavy bleeding after birth. Participants across the three countries reported several common steps for detecting PPH. First, the provider examined and observed the woman after birth for any signs of vaginal bleeding heavier than expected. To determine this, they used visual estimation, which was reported as looking at the blood pooling on the bed under the woman or at materials used to absorb or wipe away the blood. The staff counted the number of maternity pads, gauzes, wrappers (two yards of cotton material that can soak nearly 500 ml of blood), and/or “linen savers” (disposable, waterproof mats to protect the bed linen from fluids). Occasionally, they also collected blood in containers (e.g., kidney basins). Notably, no calibrated containers were available or used to quantify blood loss. Use of any tools or equipment for blood loss estimation varied by country and different equipment for blood loss estimation are reported in Table 3. In addition to visual estimation, participants relied on vital signs, particularly blood pressure and pulse rate, and other signs and symptoms of haemodynamic instability.

**Table 3:** Summary of blood loss thresholds and vital sign changes for detection of postpartum haemorrhage according to healthcare providers (*N* = 45) in Kenya, Nigeria, and South Africa.

Country	Primary PPH^a^ following vaginal birth	Severe PPH following vaginal birth	Vital sign Changes	Protocols/ guidelines/ Standard operation procedure followed	Method used for PPH detection and equipment used for quantifying blood loss for PPH at different hospitals
Kenya	≥500 ml blood loss within 24 h	≥1,000 ml of blood loss	Any blood loss that has the potential to produce haemodynamic instability should be considered a PPH^a^.	International guidelines (World Health Organization) Local guidelines provided by the Ministry of Health	Visual estimation on and underneath bed. Equipment/ supplies: *Linen/ Maternity pads/ Gauze If 4-5 pads are used in an hour after childbirth that is considered PPH* *Measuring jar*
Nigeria	≥500 ml blood loss within 24 h following childbirth any bleeding that can affect the PCV^a^ more than 10%	Threshold for blood loss ranges from 1,000 ml to 2,500 ml	Any blood loss that has the potential to produce haemodynamic instability (decreased BP, increased PR, rapid breathing) should be considered a PPH^a^. The woman will exhibit instability in physical state (pallor, dizziness, fainting, shock)	Royal College of Obstetricians and Gynaecologists (RCOG) Local guidelines adapted from international guidelines	Visual estimation on and underneath bed. Equipment/ supplies: *Wrappers, Sanitary or maternity pads or under pads* *Gauze;* Kidney dishes or measuring jug. Waterproof delivery mat
South Africa	≥500 ml blood loss within 24 h	≥1,000 ml of blood loss	PR^a^ and BP^a^ are normal until blood loss exceeds 1000 ml; tachycardia, tachypnoea; fall in BP^a^ with 1000-1500 ml blood loss; rapid PR; worsening tachycardia, tachypnoea, and exhibiting instability in physical state (pale, dizziness, fainting, shock)	National guideline adapted from international guidelines for PPH (such as RCOG) ESMOE algorithm	Visual estimation of blood loss on and underneath bed. Equipment/ supplies: *Maternity pads + Linen savers (One linen saver estimated to soak about 300-500 ml blood.*

^a^ PPH, postpartum haemorrhage; PCV: packed cell volume; BP, Blood pressure; PR, Pulse rate; RCOG, Royal College of Obstetricians and Gynaecologists; ESMOE, Essential Steps in the Management of Obstetric Emergencies.

The steps to PPH detection were similar across the three countries and mainly midwives and nurses would first detect a PPH. Most women in labour were attended by midwives across three countries; however, in Nigeria doctors also attended women due to staff shortages. In South Africa, more participants described relying on vital signs to detect PPH than did participants in Kenya and Nigeria. The length of time women was monitored postpartum for PPH varied, in Kenya and Nigeria, participants reported monitoring women after birth for two hours, at 30-minute intervals; the South African participants did not report duration for monitoring.

### Challenges of using visual estimation of blood loss

Participants discussed challenges they experienced using visual estimation to accurately assess blood loss across four critical areas, detailed below: (1) inaccurate, time-consuming, and misleading; (2) threshold of 500 ml may not be appropriate for all women; (3) shortage of equipment specific for PPH detection; and (4) incomplete communication of PPH signs between staff and women.

#### Challenges of visual estimation: inaccurate, time-consuming, and misleading

Most participants noted the subjectivity, inaccuracy, and unreliability of visual estimation of blood loss, especially “*a tendency to underestimate blood loss” [Head of Obstetrics and Gynaecology, Nigeria].* Some participants reported that accurate visual estimation of blood loss required years of experience: “*…if you have worked for long in maternity, just looking at the amount of blood that has come out, you can tell when this is not normal*” *[Nurse-Administrator, Kenya].* More junior providers found visual estimation challenging, as reported by this doctor:

*"I think quantifying blood loss volume, for me, is a big struggle. I'm not aware of any system that has concrete, there is a sort of pad is 60 ml, or if it's eight by eight centimetres…but I just find it really difficult to quantify the volume of blood loss in detecting PPH. [Intern Doctor, South Africa]*.

Healthcare providers perceived monitoring a woman after birth as time-consuming, and particularly challenging during staff shortages and a high volume of emergency cases:

*"You get busy with other [emergency] patients, and you have to go back and see what is happening so busy-ness and shortage of staff is always an issue.” [Advanced midwife, South Africa]*.

Visual estimation was likewise limited in that it did not account for women with PPH but no or limited visible blood loss:

*"…For concealed haemorrhage, you may not quantify the extent of blood loss.” [Head of Obstetrics and Gynaecology, Nigeria]*.

Healthcare providers typically counted blood-soaked wrappers or maternity pads to help estimate blood loss. However, this was complicated by women often discarding maternity pads without informing the staff or not considering urine output on the pads or wrappers while describing total blood loss. While discussing the limitation of visual estimation, a nurse in Kenya emphasised the importance of a calibrated option for measuring blood loss:

*“I ensure I have normal saline [container] when it has calibration of 250–500 ml, personally I fill the blood inside there, then I measure” [Nurse, Kenya]*.

Healthcare providers also relied on changes in vital signs to complement visual estimation of blood loss. Healthcare providers claimed that such approaches could detect severe PPH, not mild or moderate PPH:

*"… but you know these* [visual estimation and assessment of vital signs] *will only determine severe forms of postpartum haemorrhage, and it is not going to detect mild postpartum haemorrhage” [Head of Obstetrics and Gynaecology, Nigeria]*.

#### Challenges of visual estimation: threshold of 500 ml may not be appropriate for all women

Healthcare providers argued that for women with medical conditions like anaemia or other risk factors, the threshold of 500 ml for blood loss for PPH detection was inappropriate. They also frequently mentioned how PPH detection was delayed because of incomplete medical records from women who were unbooked to birth in the hospital, lack of baseline hemoglobin (Hb) status for women who did not access antenatal care or how much blood had been lost before a referred woman with PPH arrived from a lower-level health hospital:

*"Those patients who are un-booked, or they are coming for the first time during the labour…difficult to assess their PPH, especially those with background anaemia.” [Doctor, Nigeria*].

Therefore, healthcare providers emphasised the importance of a standard and objective measure of blood loss for PPH detection, which when combined with knowledge of the woman's history and haemodynamic status, could lead to prompt action if warranted.

#### Challenges of visual estimation: incomplete communication of PPH signs between staff and women

Several participants reported that the lack of clear communication between healthcare providers and women about PPH signs sometimes hindered PPH detection using visual estimation. Due to staff shortages, midwives or nurses often could not continue monitoring the woman for bleeding and had to leave to attend another woman:

*“After a delivery, you are supposed to stay on and monitor the mother. The nurse may be alone, and she has moved to another patient.” [Nurse-in-charge, Kenya]*.

Participants in South Africa and Nigeria mentioned that they also encouraged the women to intermittently massage their own uterus postpartum, especially where monitoring capacity was poor, and asked the woman and her family to report if they noticed any heavy bleeding. However, participants reported that women across three countries had limited understanding of what amount of postpartum blood loss was heavy. However, some participants agreed that communication between staff and women was not clear enough, which could hinder PPH detection:

*"I think it's communication… if you don't communicate the patient just goes to the postnatal ward where she continues PPH-ing and there wasn't proper handover or communication then it* [PPH] *can get missed.” [Advanced midwife, South Africa]*.

### Primary management of PPH

Healthcare providers explained how they managed PPH according to PPH protocols or guidelines. They also highlighted the importance of teamwork and the challenges they experienced at the hospital level during PPH management.

#### Use of guidelines for PPH management

Although most participants reported that they used local, national, and/or international guidelines for PPH management, a few midwives or nurses were unaware of such guidelines. National guidelines were from each country's Ministry of Health, and participants in Kenya and South Africa reported using the WHO Guidelines for PPH (10, 21, 42), whereas participants in Nigeria used the United Kingdom, Royal College of Obstetricians and Gynaecologists (RCOG) guidelines (43). Some hospitals in Kenya and South Africa also had their own hospital-based Standard Operating Procedures for PPH management, participants in Kenya often attended government-sponsored BEmONC training and used Kenyan BEmONC guidance (44), and those in South Africa used the South African Department of Health Maternity Care Guidelines (45) and ESMOE training materials and algorithms (40).

While discussing the accessibility of guidelines, several participants in Nigeria mentioned that they only had online access to guidelines and reported that using them was inconvenient as they had to download and print them. In Kenya and South Africa, participants mentioned having access to the hard copy of the guidelines, which were kept at their workstations. All participants mentioned that the tertiary hospitals had pictorial posters of PPH management in the labour room or ward, in the delivery room, in the nurses' station, and in the operating theatre. However, for secondary-level hospitals, posters might not be available, according to a few participants.

Participants stated that guidelines were helpful in an emergency like PPH when all staff were required to act promptly and synchronously:

*”… PPH is normally an emergency and when you are dealing with an emergency, …. It makes a lot of sense when you have such guidelines, and everybody has gone through them and appraised them so that actual management team works in synchronised manner.” [Gynaecologist, Kenya]*

However, participants provided mixed responses while discussing how often they used guidelines. Several participants, mainly more experienced doctors, stated that they did not need to revisit guidelines as there was nothing new added to the existing guidelines, and they knew the steps by heart, working on autopilot “like a reflex”. They claimed that those existing guidelines were not updated or did not add any new evidence; therefore, they were more beneficial for new or junior staff. Several midwives and junior doctors reported that they used the guidelines more often to internalise the steps. Furthermore, a few midwives and nurses in South Africa reported that managers never reinforced them to use the guidelines.

Participants perceived that they needed more updated guidelines to improve their knowledge and practice for PPH management. In addition, participants in Nigeria and Kenya revealed that local guidelines were not updated in line with new evidence and the international guidelines were difficult to follow as they were not context-specific. For example, not all drugs recommended in the guidelines were readily available at hospitals in resource-poor areas, and therefore, healthcare professionals adapted their practice according to their context: “*We use WHO guidelines. But we have tailored these to the basics. We sometimes substitute the drugs of choice as per the guidelines, with what we have available in the hospital”. [Doctor, Kenya]*.

#### Steps of PPH management

Participants provided detailed steps of clinical interventions for PPH management. Although they applied similar interventions across three countries, there were substantial differences in how the steps were applied by different health cadres, as follows. Upon detecting PPH, the attending midwife or nurse would call for help (from other midwives or doctors), while applying uterine massage and examining for any clots, retained birth products, and lower genital tract trauma. Meanwhile, midwives or nurses would secure two intravenous lines for administering uterotonics and fluids and assess vital signs. Uterine massage and uterotonics (oxytocin and/or misoprostol) would be given together, mentioned mostly by participants in Kenya and South Africa (Nigerian participants did not explicitly mention if they administered uterine massage and uterotonics together). If bleeding continued after administration of oxytocics, the doctor may prescribe TXA (if available) and would typically administer TXA only as a “*last resort*”. At the same time, blood samples would be collected for complete blood count, PCV, and grouping and matching for possible blood transfusion.

Midwives and nurses in Kenya and Nigeria reported that they felt confident performing most of the steps, except for administering TXA and performing upper genital tract examination. Usually, a doctor would order and administer TXA and perform the genital tract examination. A few participants in Nigeria mentioned that besides doctors, experienced midwives or nurses could perform genital tract examinations. Nigerian participants also mentioned that due to the higher cost and unavailability of the TXA, the woman's relatives usually purchased it.

In South Africa, most participants mentioned that they follow the ESMOE algorithm for PPH management. Although PPH management steps were similar in Kenya and Nigeria, the person that initiates PPH treatment may vary for healthcare providers working in tertiary and district hospitals in South Africa. Midwives working at the district hospital in South Africa reported that they would call for help from other midwives and nurses upon detecting PPH and would play an active role in initiating uterine massage, administering uterotonics and IV fluids without a doctor's involvement. They would also inform the doctor on call, but they usually would not wait for doctor's arrival to initiate PPH treatment. According to an advanced midwife at the district hospital, she could order for and administer TXA if bleeding continued. On the other hand, a few participants working at the tertiary hospitals provided mixed views regarding roles and involvement of different health cadres in PPH management. A few participants reported that after detecting PPH, a midwife would call for help from other midwives and nurses, and they would also inform the duty doctor. Together, the midwives and nurses' team would initiate PPH treatments including administering massage, uterotonics, and IV fluids. The doctor would usually prescribe and/or administer TXA and perform the genital tract examination for any trauma or tears. However, a few participants including midwives and doctors mentioned that nurse-midwives would not administer any drugs without a doctor's presence or orders over phone. Usually, a doctor would join the midwives' and nurses' team to initiate treatment of PPH.

#### Importance of teamwork in PPH management

Participants in all countries agreed that “*all hands must be on deck”* and teamwork was key to successful PPH management, describing it as *“a collective effort rather than an individual effort, so you look for everybody's help”.* Despite this, participants identified several challenges to teamwork. Not all team members had equal levels of knowledge and skills, therefore the team cannot always perform in an organised manner. Miscommunication or lack of communication between midwives and nurses as first responders and doctors were further key barriers to teamwork reported by the participants in Kenya and South Africa. Furthermore, having team members who were well acquainted was crucial to teamwork:

*“Maybe what would work better…if we can form a team which is known by everybody, as PPH team, so just in case of a PPH this team can be called”. [Nurse, Kenya]*.

In all countries, staff shortages were a hindrance to team formation and performance. Several participants also indicated that team members might have other emergencies requiring their attention, and as such, not all staff could respond to a PPH immediately.

*“I can say in this facility we usually have a lot of clients, so you will find especially with the duty allocation, someone is allocated in first stage of labour, another person is allocated with post-op patients, so this divides the nurses and when you actually want them to work as a team, they will be concentrating on their areas of allocation, instead of actually coming and converging to this one patient…” [Doctor, Kenya]*.

### Challenges or barriers to primary management of PPH

Common challenges regarding PPH management were reported across the three countries, and were categorised under three domains: (i) challenges within the hospitals; (ii) from the referring hospital; and (iii) at the women's level.

#### Challenges within the hospitals: shortage of staff with appropriate skills

Staff shortages were identified by participants in all countries, which resulted in delayed PPH detection, and consequently delayed PPH management interventions. In busy labour wards, staff shortages become more acute due to low staff-to-women ratios, which limits how closely women were monitored during intra- and postpartum. A few participants explained that some midwives or nurses who were first-responders to PPH did not have expertise in detecting internal bleeding, because it was not in their scope of practice. Furthermore, junior or new medical officers lacked knowledge and skills for early PPH detection, as one doctor explained: “ *I think it is not having enough knowledge [among new staff] on the PPH…Because maybe sometimes we are unable to quantify the amount of blood lost, then we say this is PPH, or this is just normal blood loss.” [Doctor, Kenya]*.

In all countries, this impact was exacerbated for midwives and doctors by the limited opportunity for in-service training for PPH management, which was viewed as desirable, as one nurse-midwife explained:

*“Continuous training…because training is going to empower you, you understand. You can never have enough training, especially for stuff like PPH; it is something that will always be there because it's one of the complications of maternity unit emergencies so, more training, training, training and training.” [Nurse-Midwife, South Africa]*.

During busy times in settings where doctors are required to confirm midwives’ preliminary diagnosis of PPH and initiate treatment, they may have to prescribe PPH interventions without examining the woman themselves: “*…we're always on guard. So sometimes I think we may even over treat. ….. there isn't a doctor attending and who's not there to offer his physical help, and it's done over the phone.” [Head of Obstetrics and Gynaecology, South Africa]*.

#### Challenges within the hospital: lack of essential resources for PPH management

Essential drugs and equipment and availability of resources to store resources were often missing or compromised across all countries. In Nigeria, participants reported regular stocks of oxytocin but also reported that disruptions in the cold chain during procurement or storage may have compromised drug quality. Moreover, participants in Nigeria believed that not all brands of oxytocin were equivalent, and sometimes they received poor quality oxytocin:

*“…Not available here like the Soxyn, Novartis [Oxytocin brand] is good, but it is not available because it is expensive actually. But the ones [Oxytocin brand] we have are not effective…. You need higher doses instead like of 10IU. You need additional 3 more vials.” [Doctor, Nigeria]*.

Participants in Kenya reported using misoprostol, but they also experienced shortages, as one nurse described that they *“…have misoprostol today, but the next day it is all gone.” [Nurse, Kenya]*. In Kenya, participants reported using TXA, but that high costs impacted regular supplies. In Nigeria, participants reported using TXA, but that women's relatives had to purchase it from pharmacies outside the hospital.

Sometimes women who are unbooked present at the hospital without health records, such as blood group information or history of anaemia. This results in delayed treatment initiation while blood samples for grouping, matching, and hemoglobin level are drawn and examined; as not all hospitals have essential resources (laboratory, laboratory technicians) 24/7 to do this. Furthermore, arranging blood for rare blood types was more challenging and further delayed PPH management. In some cases, the woman's relatives may be asked for blood donations to save her life, but even then, delays can ensue, as described by a nurse:

*“…if you send the relatives to go for [blood] grouping and cross-matching, they will stay long before they come back to bring blood for transfusion, and sometimes the staff there is not around or there is more people [long queue] in the blood bank”. [Nurse, Nigeria]*.

Emergency blood products such as fresh frozen plasma and platelets were not readily available at many hospitals, which not only delays treatment but may lead to development of Disseminated intravascular Coagulation.

Due to the lack of essential resources (particularly lack of bed capacity, lack of staff, during over-flow of patients) at hospitals, healthcare providers often refer women with PPH to another hospital, which may take hours to reach. Ambulances or alternative transportation are not typically readily available, and the receiving hospital may not have all the resources (e.g., staff, drugs, medical history of patient) ready to provide appropriate care.

#### Challenges from the referring hospital: late referrals

Participants stated that managing PPH was usually more challenging for women who were referred by another hospital or from the community, compared to women who attended antenatal care and had given birth in the same hospital, as the women who were referred may not have received any care or inappropriate care. One doctor described:

*“…late presentation is also one of the issues and challenges that we have especially from those who are coming from outside because most times they would have gone to other peripheral hospitals, 2 or 3 other place peripheral hospitals and patient has been mismanaged before they now present to you. So, when they come to the hospital, they come in severe PPH.” [Doctor, Nigeria]*

Women who were referred from other hospitals or the community may have incomplete patient histories, leading to further delays in PPH management. Lack of proper communication with the referral hospital was identified as a challenge. One participant suggested that a complete referral letter would be a solution to minimise the challenge:

*“… we don’t really have a smooth communication where we will be referring the patients to so that when they get to that other facility, it will not be the case of starting from the beginning. The only way we do is we actually give a written referral. Hence, if there is a way, we can breach that gap so that once she [the woman] arrives, the person receiving her has a basic history because we were able to communicate, I think it will go a long way in helping. [Consultant of Obstetrics and Gynaecology, Nigeria*].

#### Challenges with obtaining consent for genital tract examinations

Midwives and doctors across three countries reported that some women refuse genital tract examination due to discomfort or to preserve their privacy and modesty, as one nurse described: “*…some of the patients will deny [genital tract examination] because of that privacy, they don’t want too much of exploration. [Nurse, Nigeria].* When women refused, midwives highlighted the need for clear communication about the purpose and importance of the examination.

### Comparing current practices to WHO recommendations for PPH management

In Table 4, we use the WHO recommendations for PPH management and compare the participants' responses to explore areas of convergence and divergence between recommended and actual practices. Table 4 depicts that with a few exceptions, most participants' responses to primary management of PPH were aligned with WHO Guidelines (10, 21, 42). Differences were mainly around lack of resources to maintain the cold chain for oxytocin, unavailability of TXA due to high cost, performing invasive procedures as the primary response (UBT, bimanual compression, speculum exam for genital tract trauma), lack of referral protocols, and lack of simulation-based training.

**Table 4:** WHO recommendations on management of postpartum haemorrhage compared with interview participants’ qualitative responses to the questions: *think back to the last time that a woman under your care, with a vaginal birth, had a PPH. Could you describe what happened, what did you do and why did you do it?.*

Topic	WHO recommendations^a^	Kenya	Nigeria	South Africa
Uterotonics	Intravenous oxytocin is the recommended uterotonic drug for the treatment of PPH. If intravenous oxytocin is unavailable, or if the bleeding does not respond to oxytocin, the use of intravenous ergometrine, oxytocin-ergometrine fixed dose, or a prostaglandin drug (including sublingual misoprostol, 800 µg) is recommended.	Most participants mentioned using oxytocin and misoprostol for PPH treatment, but sometimes there are stock out of uterotonics. Maintaining cold chain for oxytocin was identified as a challenge.	All participants mentioned using oxytocin for PPH treatment, but sometimes there are stock outs. A few participants mentioned giving misoprostol if oxytocin does not stop the bleeding. Maintaining cold chain for oxytocin was identified as a challenge.	All participants mentioned using oxytocin for PPH treatment and several mentioned of using misoprostol. Sometimes there are stock out of uterotonics.
Intravenous fluids	The use of isotonic crystalloids is recommended in preference to the use of colloids for the intravenous fluid resuscitation of women with PPH.	All participants mentioned starting IV fluids as a primary response and IV lines were put in on admission	All participants mentioned using IV fluids for PPH treatment. None mentioned when they set the IV lines on women.	All participants mentioned using IV fluids for PPH treatment and the IV lines usually set up early before veins collapsed.
Tranexamic acid	Early use of intravenous tranexamic acid (within 3 h of birth) in addition to standard care is recommended for women with clinically diagnosed postpartum haemorrhage following vaginal birth or caesarean section (10 mg slow IV push over 10 min)	Nearly all participants were aware of benefits of TXA but did not have regular access to TXA on maternity ward because of its high cost. Viewed as only to use in severe cases i.e., refractory PPH and only doctors could administer the TXA. No mention of dose, route, or time.	Nearly all participants were aware of benefits of TXA but did not have regular access to TXA on maternity ward because of its high cost. Viewed as only to use in severe cases i.e., refractory PPH, and only doctors could administer the TXA. No mention of dose, route, or time used. Although it was incorrect, a few mentioned it was most effective when given within 15 min of childbirth	All participants were aware of benefits of TXA (Cyclokapron) and did not have regular access to TXA on maternity ward because of its high cost. Viewed as only to use in severe cases i.e., refractory PPH, and only doctors could administer the TXA. No mention of dose, route, or time
Uterine massage	Uterine massage is recommended for the treatment of PPH.	Most participants mentioned that uterine massage was currently used as first line treatment for PPH, and a few mentioned applying uterine massage and administering oxytocin simultaneously. Several participants mentioned administering bimanual compression for primary PPH management.	Most participants mentioned that uterine massage was currently used as first line treatment for PPH, and a few mentioned applying uterine massage and administering oxytocin simultaneously. A few participants mentioned sometimes they taught women how to massage the uterus. A few participants mentioned administering bimanual compression for primary PPH management.	All participants mentioned that uterine massage was currently used as first line treatment for PPH, and several participants mentioned that uterine massage administered simultaneously with oxytocin. A few participants mentioned administering bimanual compression for primary PPH management.
Clinical protocols for treatment	The use of formal protocols by hospitals for the prevention and treatment of PPH is recommended.	Most participants mentioned using hospital-based standard operating procedures (SOP) for PPH management and having pictorial posters in the labour ward. These SOPs were developed and provided by the Ministry of Health.	Several participants mentioned being aware of clinical protocols of PPH management that were adapted from international guidelines but could not access easily (online version only). Pictorial posters of PPH management steps were available in the labour ward.	All participants mentioned using hospital-based SOPs for PPH management and having pictorial posters in the labour ward. National guideline for PPH management was adapted from international guidelines for PPH.
Clinical protocols for referral	The use of formal protocols for referral of women to a higher level of care is recommended for hospitals.	Participants did not report having access to clinical protocols on the labour ward for referral but thought this would be helpful because many lower-level facilities refer women without complete medical history. All mentioned receiving women with PPH from lower-level facilities, since they worked in tertiary level hospitals.	Participants did not report having access to clinical protocols on the labour ward for referral but thought this would be helpful because many lower-level facilities refer women without complete medical history. All mentioned receiving women with PPH from lower-level facilities, since they worked in tertiary level hospitals. A few mentioned referring women to other hospitals if theatre or beds were unavailable.	Participants did not report having access to clinical protocols on referral in the labour ward but reported his would be helpful as many lower-level facilities refer women without complete medical history. All mentioned receiving women with PPH from lower-level facilities, since they worked in tertiary and District level hospitals. A few mentioned referring women to other hospitals if theatre or beds were unavailable.
Training on PPH treatment	The use of simulations of PPH treatment is recommended for pre-service and in-service training programmes	Over half of the participants in Kenya reported attending an in-service, national Basic Emergency Obstetric Care which has a simulation component and most participants expressed desire more training.	Most participants had not had any in-service on PPH management since their pre-service education, but desired more training	Most participants attended d, 3-day ESMOE^a^ training which has a simulations component. Most participants expressed desire for more training

^a^ ESMOE, essential steps in the management of obstetric emergencies.

## Discussion

We conducted this formative study to inform the development of the E-MOTIVE trial, with an aim to explore knowledge and practices of PPH detection and primary management among healthcare providers in Kenya, Nigeria, and South Africa. We identified critical challenges and opportunities to improve PPH detection and management in hospital settings, including improved in-service training for PPH, more reliable and accurate approaches to PPH detection, improved guideline adaptation and use in clinical settings, enhanced teamwork and communication, streamlining referrals, and improved reliable access to resources.

Participants noted the lack of in-service refresher training as undermining their clinical confidence, evidence from other LMICs shows that healthcare providers who do not attend any refresher training after starting their professional careers have low confidence in treating women with PPH (46–48). There were limited in-service training opportunities for healthcare providers across the three countries, indicating knowledge and skills gaps on current evidence-based clinical PPH detection and management (47, 48). Considering the unequal knowledge and skills among different individuals and cadres of maternity healthcare staff, participants' willingness to receive multidisciplinary simulation training would allow them to improve their knowledge and skills (46, 47, 49). Therefore, when implementing a new PPH care bundle like E-MOTIVE, it is important to provide multidisciplinary simulation training for different cadres of maternity healthcare staff to minimize the know-do gaps.

For PPH detection, the healthcare providers across three countries mostly followed the blood loss threshold of ≥500 ml (and ≥1,000 ml for severe PPH) through visual estimation and assessment of vital signs recommended in international PPH guidelines (5, 10). However, we also found that participants questioned the applicability of the blood loss threshold (≥500 ml) for all women, particularly in sub-Saharan Africa with its’ high prevalence of women with anaemia (50). These findings suggest that existing guidelines concerning blood loss thresholds for PPH detection and management may need context-specific adaptations (14, 51), with more attention to Hb measurements during pregnancy and on admission in labour to enhance risk assessment.

Participants in our study also asserted that blood loss estimation using non-calibrated kidney dishes or by quantifying the number of blood-soaked materials (linen savers, maternity pads, wrappers) was complex and produced unreliable results. While these visual estimation approaches may be considered practical and cost-effective, participants criticised their subjective nature. In line with previous studies, participants in our study claimed that the visual estimation was time-consuming and often gave inaccurate estimation (14, 17). Similar to other study findings from LMICs, healthcare providers had limited resources (skilled staff, beds, medical supplies) where one staff member often dealt with multiple obstetric emergencies at once. As a result, they often missed PPH, a finding mirrored in other LMICs studies (14, 17). Along with limited resources, unclear communication between staff and women regarding recognition of PPH often negatively affected PPH detection, as reported in previous studies (14, 48). These findings suggest that healthcare providers in the study contexts may find that more objective methods of blood loss measurement using calibrated tools may be highly acceptable. Midwives' and doctors' acceptability of calibrated tools such as a drape (calibrated blood collection bag) has been similarly reported in other contexts as more accurate, objective, and feasible to implement (16, 52–54). Further research including the E-MOTIVE trial is needed to explore the feasibility and acceptability of calibrated drapes for both providers and women to develop a standard, more objective obstetric practice for PPH detection in these study settings.

While first-line PPH management was described similarly by our participants in Kenya and Nigeria, we found that TXA was considered unavailable and expensive. Doctors administered TXA as a “*last resort*” and the woman's relatives might need to purchase it outside the hospitals. Our findings also revealed that midwives and nurses who attended births and managed PPH were often not allowed to administer TXA. Hospitals where emergency obstetric care is provided need to have the necessary supplies, training, and clinical protocols for the safe administration of TXA, given its value as a life-saving intervention. Given that a large randomised control trial (WOMAN) demonstrated clear mortality benefits with early use of TXA (55), and WHO recommends early administration of intravenous TXA for all women with PPH (22), it is concerning that this recommendation has not yet translated to practice. Since administration of TXA is a key component of the E-MOTIVE bundle, the trial will provide the opportunity to explore the feasibility and acceptability of using TXA at scale.

Despite often being first responders to PPH, not all midwives and nurses could proceed with all primary PPH treatments; this was most commonly reported in tertiary-level hospitals in South Africa. Some South African participants stated that first responders in the tertiary hospitals often were not allowed or trained to administer TXA or perform genital tract examination without a doctor's prescription and supervision, and such gaps in skills often contributed to poor communication within the team. Although PPH management demands immediate actions and “all hands-on deck”, task divisions based on profession creates unequal expertise among staff, limits effective communication, and can delay response in an emergency (48, 56, 57). Considering the shortage of resources, including skilled staff, upskilling first responders to obstetric emergencies may help to provide more timely care (48).

Given the complexity of PPH management, the participants emphasised effective teamwork among nursing, midwifery, doctors, and obstetricians (58). They also highlighted that all levels of maternity staff should have similar skill-sets, including skills that may not always be prioritised: communication, cooperation and coordination (59). Evidence from Nigeria and the United States suggest that team-based, multi-professional simulation training benefits healthcare providers of different cadres to develop shared mental models that helped them to work quickly, efficiently, and harmoniously as a team to manage PPH cases (49, 60). Team-based multi-disciplinary training also helps to challenge existing hierarchical relationships, build confidence, clarify roles and task management, improve provision of woman-centred care and communication, and encourages healthcare providers to rehearse, repeat and reinforce their knowledge (49, 60). Besides training opportunities, evidence from other LMICs suggests that communication among staff and their performance could be improved through active clinical supervision, mentoring, and leadership by senior health professionals (56). Finally, the comparison between current clinical practices of PPH management across three countries and the WHO recommendations for PPH management indicates that there are inconsistencies in administering different interventions. A PPH care bundle such as E-MOTIVE may offer a possible solution to address inconsistencies and improve adherence to guidelines and good quality care (3, 20).

The findings of this qualitative study are to inform the design of the E-MOTIVE trial and implementation strategies. Following a rapid analysis of the data presented in this paper, we brought the qualitative findings to teams from each country to discuss in stakeholder consultations workshops, which consisted of the E-MOTIVE research team, maternity care providers working in the study sites, and other key stakeholders (20). We discussed the challenges raised by the research participants and discussed potential solutions to address these challenges within the context of the E-MOTIVE trial. These solutions took the form of potential implementation strategies to be delivered alongside the E-MOTIVE bundle in the trial (e.g., training, champions, audit, and feedback). The implementation strategies developed as a result of these workshops have since also been tested in adaptive cycles in each country to explore the feasibility, fidelity, and acceptability of the intervention (20). The intervention design and key findings from the stakeholder consultation and workshops and adaptive cycles will be discussed in detail in subsequent publications. If the E-MOTIVE trial is effective, we plan to use results from this formative study as well as the forthcoming process evaluation to develop strategies to improve the sustainable implementation and scale-up of the E-MOTIVE bundle, including how to best address system-level issues.

### Limitations and strengths

An important limitation of this study was that most interviews were conducted using virtual platforms during the COVID-19 pandemic. Participants who choose Zoom sometimes needed to switch to a phone call because of connectivity issues. This affected the flow of the interviews; hence, affected data quality in a small sample of the interviews. Furthermore, interview findings may be subject to social desirability bias due to how questions were asked or the professional backgrounds of the researchers (mainly medical professionals) conducting the interviews. Different professional cadres, such as nurses and midwives, or junior doctors, may have answered questions with socially desirable responses. To minimise this limitation, regular discussions occurred between the wider team and interviewers following two to three interviews and any issues related to leading questions and missed opportunities of probing were discussed. Finally, all interviews were conducted in predominantly higher-level hospital-based settings, and the findings may not be transferable to lower-level hospitals with fewer resources and different staff mixes. For example, in lower-level hospitals, midwives may have more competencies and autonomy to detect and manage PPH, compared to the study settings.

The major strengths of our study included our design and analysis approach, which provided results that are credible, transferable, dependable, and confirmable to ensure rigour (61). Our participants came from a wide variety of professional different work environments, and with different opportunities and diversity in cultures. This wide range of participants maximized variability in terms of representing the various perspectives of healthcare providers who detect and manage PPH in Sub-Saharan Arica. Furthermore, using a theory-based interview topic guide and the consistency of semi-structured questions in all study sites enhanced the richness of collected data. A consistent template was used for transcription to obtain consistency between the transcripts. Furthermore, all participants were offered the opportunity to review their interview transcripts for validation; this approach enabled the researchers to verify the accuracy of the transcripts and their interpretations. All members of the formative research team actively were involved in the analysis process and confirmed the accuracy of the analysed data. Moreover, a systematic and transparent approach of data analysis to describe healthcare providers' experiences, as well as their demographics and study settings, may enable readers to extrapolate the findings within other similar settings (62). Researchers were reflexive and aware of their knowledge and experience, to minimize their influence on data collection, analysis, and interpretation of research results.

## Future research directions

The aim of this formative study was to inform the development of the E-MOTIVE bundle intervention and implementation strategies. Bundle implementation includes leadership, teamwork, feedback, and communication, including communication between providers and the woman and her family, and these feasibility, acceptability and effectiveness of these strategies needs to be explored in future research. Moreover, the health facilities included as study sites in this formative research were reflective of the types of health facilities appropriate for the E-MOTIVE trial; therefore, they needed to be sites where women with PPH could be cared for without onward referral. If the trial shows that the E-MOTIVE bundle is effective in these settings, then a critical next step will be exploring implementation and scalability in other contexts, including lower-level health facilities. More research will be needed to consider best approaches to E-MOTIVE bundle implementation into routine clinical practice, as well as scalability and sustainability. Future research should explore the broader health systems factors that require substantial quality improvement, as well how to best integrate the E-MOTIVE bundle into broader quality of care initiatives, such as strengthening the health workforce, improving provision of respectful maternity care (including labor companionship), and improving management of other serious maternal complications.

## Conclusion

Our study identifies critical needs to address context-specific barriers of early and timely detection and management of PPH in high-burden settings. The findings of this study reflect provider perspectives on the inaccuracy and unreliability of visual estimation of blood loss, resulting in underestimation and delayed detection of PPH. This indicates the important need to develop and evaluate more objective and reliable approaches to PPH detection. Multi-disciplinary simulation training to improve team communication, coordination, and collaboration and consequently to improve clinical practices of PPH detection and management, as well as ensure respectful maternity care would be a practical approach to support healthcare providers in low-resource settings and may be acceptable in Kenya, Nigeria, and South Africa. Our research findings will be used to develop evidence-informed implementation strategies, such as improved in-service, simulation training, and more objective blood loss measurement tools as part of the E-MOTIVE trial.

## Data Availability

The original contributions presented in the study are included in the article/**Supplementary Materials**, further inquiries can be directed to the corresponding author/s.
