# Supplementary material for: Detection and management of postpartum haemorrhage: Qualitative evidence on healthcare providers' knowledge and practices in Kenya, Nigeria, and South Africa
Source: Front Glob Womens Health. 2022 Nov 18;3:1020163. doi: 10.3389/fgwh.2022.1020163 (PMC9715762; doi:10.3389/fgwh.2022.1020163)
Supplement: Supplementary file 1 [file Datasheet1.docx]

Appendices

## Appendix 1: In-depth interview guide

**In-depth interview guide: healthcare providers**

Thank you very much for taking the time to speak to me today. My name is [interviewer name], and I am one of the E-MOTIVE study team members. Before we begin, can I please confirm that you have received a copy of the study information sheet and consent form?

As a reminder, this study aims to explore how postpartum haemorrhage (PPH) is currently detected and managed in facilities such as this. We are interested in hearing your views and experiences about what currently happens in practice, and what factors influence how PPH is detected and managed. There are no right or wrong answers. Everything you say will be treated confidentially and will not be shared with any of your colleagues, or anyone outside of the E-MOTIVE study team. You are free to answer in as much or as little detail as you wish, to skip over any questions you do not wish to answer, and to pause or stop the interview at any time if needed.

This interview will take approximately one hour- depending on how much you have to say. Can I please check you are free at the moment to talk for this amount of time?

I would also like to please record our conversation- so that I can capture your responses accurately, and so that I can listen to you rather than take many notes. Can I confirm you are happy for me to start recording?

Great. Thank you.

| Note to interviewer:   1. Please ask all questions in **BOLD**. 2. Wait before asking the *prompt questions (in italics)* until after the participants has responded to the question in bold. 3. Use the *prompt questions* to get more detailed information about **why** these personal views influence the detection and management of PPH, in addition to how PPH is detected or managed, and who does what and when.   **During the interview**, please keep track of what’s said about any of the following interventions mentioned by the participant when managing PPH:  ***Massage of uterus*** 🞎 ***Use of Oxytocics*** 🞎 ***Use of Tranexamic acid*** 🞎 ***Giving IV Fluids*** 🞎  This information is required for questions about the E-Motive bundle. |
| --- |

**Section 1: Background**

*We would like to start with understanding a bit about your role in your current job and the facility where you work:*

*We would like to start with understanding a bit about your role in your current job and the facility where you work:*

1. **What is your current position**? *[Motivation/ Professional Role & Identity]*
   1. **How long have you been a [doctor/midwife/nurse]?**
   2. **How long have you been in this position at this facility?**
2. **Could you briefly tell me a bit about how the maternity ward in your facility is set-up?** *[Opportunity/Environmental Context & Resources]*

*Prompts: Are there separate rooms for different stages of labour, who works where?*

1. **Which parts of the maternity ward do you currently work on?** *[Motivation/ Professional Role & Identity]*

*Prompts: Do you work in antenatal clinic, labour, delivery, postnatal?*

- 1. **Do you currently work in any other ward?** *[Motivation/ Professional Role & Identity]*

*Prompts: if yes, what do you do?*

*Thank you for sharing. For the remainder of this interview, I would like to focus on discussing the detection and management of PPH in your facility. For the purpose of this research study, we are not interested in PPH prevention. We will be discussing PPH for vaginal birth only (not caesarean section).*

1. **Could you tell me a bit about your understanding of what postpartum haemorrhage (PPH) is?** *[Capability/Knowledge]*

*Prompts: How would you define a PPH?*

1. **Could you tell me a bit about your understanding of severe PPH?** *[Capability/Knowledge]*
   1. **How much of an issue do you think PPH is among women giving birth for health care providers and for the facility?** *[Motivation/Goals]*

*Prompts around why, explore what is that makes it an issue or not an issue*

- 1. **How much of a priority is caring for a woman with PPH in maternity?** *[Motivation/Goals]*

*Prompts: Why is that?* Is there anything at a higher priority?

- 1. **Have you received any additional training about the detection and management of PPH? In addition to that which you may have received back in [medical/nursing/midwifery] school?** *[Capability/Skills]*

*Prompts: When was the training? Where was the training? (in facility or external); what did the training cover; how was it delivered? (in classroom, online), Did you find it helpful/not helpful?*

- 1. **Do you feel any additional training is needed on PPH? If so, what would you like to receive training on?** *[Capability/Skills]*
  2. **Do you use any clinical guidelines or protocols for detecting and managing PPH in your facility? If so, which ones?** *[Capability/Memory, Attention & Decision Processes]*

*Prompt: local or national guidelines?*

*If guidelines are used: Are they displayed? If not displayed, why and where are they physically located? Is it easy/not easy to access them? How often or when would you use these guidelines/protocols? How useful are guidelines your clinical practice? Why is that?*

*If guidelines are not used? Why not?*

- 1. **Do you feel more guidelines and protocols for detecting and managing PPH are needed?** *[Capability/Behaviour Regulation]*

*Prompts: Why is that? What types of guidelines or protocols are needed?*

**Section 2. PPH Detection – Vaginal birth**

*Now I would like to ask you some questions about the detection of postpartum haemorrhage, which is the focus of this research study. Here we are focused only on vaginal birth.*

1. **Could you describe how is PPH typically detected in this facility?** *[Capability/Skills]*

*Prompts: What do you do? Who else is involved? What do they do? Where (in which rooms) is PPH usually detected? (e.g. Labour ward, delivery ward, recovery ward, post-natal ward)*

1. **Overall, is there anything that makes it difficult for you and your colleagues to detect a PPH? What gets in the way sometimes?** *[Capability/Beliefs about Capabilities]*

*Prompts:* *Is there anything that helps or makes it easier?*

1. **What factors make you suspect that a woman is having PPH?** *[Capability/Knowledge]*
   1. **How do you estimate blood loss?** *[Capability/Skills]*

*Prompt: Why do you use this method? What are the benefits and downsides of this method?*

- 1. **Can you describe an example of when it was challenging to detect PPH (in this current facility or elsewhere)?** *[Capability/Beliefs about Capabilities]*

*Prompt: Find out why and what made it challenging? What would make it easier to detect?*

**Section 3. PPH Management – Vaginal birth**

*Thank you for sharing your views on PPH detection. Now I would like to know a little more about how you manage PPH in your current facility.*

1. **Think back to the last time that a woman under your care, with a vaginal birth, had a PPH. Could you describe what happened, what did you do and why did you do it?** *[Capability/Memory, Attention & Decision Processes]*

*Prompts: How long did it take to initiate treatment? How did you make the decision to initiate treatment? Did you work alone or with a team? What did you and your colleagues do to manage the PPH? What tasks did you specifically do?*

*[If more than one step of managing PPH]: In what order did you perform these tasks?*

- 1. **In your opinion, did you work well as a team? Why or why not?** *[Opportunity/Social Influences]*

*Prompts: In general, what gets in the way of team working? What would help you work better as a team when managing a PPH?*

1. **Did you lack any resources needed to manage this PPH?** *[Opportunity/Environmental Resources & Context]*

*Prompt: time, staffing, equipment, supplies? Was everything easily available when you needed it?*

*What happens if what you need is not there or is not working?*

1. **Did you encounter any challenges or problems when managing this PPH?** *[Capability/Beliefs about Capabilities]*

*Prompts: What could be done to better support you and your colleagues to manage a PPH in this facility?*

1. **(If not covered in response to Q7) What would you and your colleagues do if a woman did not respond to initial treatment and she continued to bleed?**  *[Capability/Memory, Attention & Decision Processes]*
2. **What are some of the challenges or problems when managing a woman with refractory PPH (haemorrhage that doesn’t respond to primary measures)?** *[Capability/Beliefs about Capabilities]*
3. **What are some of the challenges or problems when managing a woman who needs to be moved to operating theatre*?*** *[Capability/Beliefs about Capabilities; Opportunity/Environmental Context & Resources]*
4. **Do you ever transfer a woman with PPH from this facility to another? If so - What are some of the challenges or problems to transferring a woman to another facility?** *[Capability/Beliefs about Capabilities; Opportunity/Environmental Context & Resources]*
5. **Does this facility ever receive transfers/referrals from lower level facilities? If so, what are some of the challenges or problems you face when receiving a woman transferred/referred from another facility?** *[Capability/Beliefs about Capabilities; Opportunity/Environmental Context & Resources]*

**Section 4. The E-MOTIVE bundle**

1. **Have you ever heard of a clinical bundle?** *[Capability/Knowledge]*

If yes, what have you heard?

If not, define as - A bundle is a set of clinical intervention treatments ALL to be used TOGETHER for every patient with a specific diagnosis.

*I would like to ask you some questions about a new specific approach to detect and manage PPH we call the E-MOTIVE Bundle.*

| **Show** the E-Motive diagram (copy below) to the participant if F2F or ask them to look at the diagram if using Zoom to be either shared on PC screen or on WhatsApp if using a mobile phone.  **READ** to the participant all the information about the bundle below. Clearly state the bundle is to be performed ALL AT ONCE not sequentially. |
| --- |

*The E-MOTIVE bundle involves performing six evidence-based strategies and interventions to detect and manage PPH, these include:*

***E*** *– Early detection of PPH, using a closed end, measuring drape. This drape is calibrated, it has visible line marking when 500 ml of blood has been lost. Once a PPH is detected using the calibrated drape, the following interventions should be performed* ***SIMULTANEOUSLY****:*

***M*** *– Massage of Uterus,*

***O*** *– administration of Oxytocic drug,*

***T*** *– administration of Tranexamic acid,*

***IV*** *– providing IV Fluids and*

***E*** *– Examination of the genital tract + escalation (as necessary).*

*These should be performed* ***SIMULTANEOUSLY,*** *without waiting for response to individual interventions. If women do not respond to the whole MOTIVE bundle, then progress to treatment for Refractory PPH. You already mentioned that you are already using some or all of these components individually in your facility to manage PPH, but we are interested in discussing how you would use all of these together, at the same time, in a bundle.*


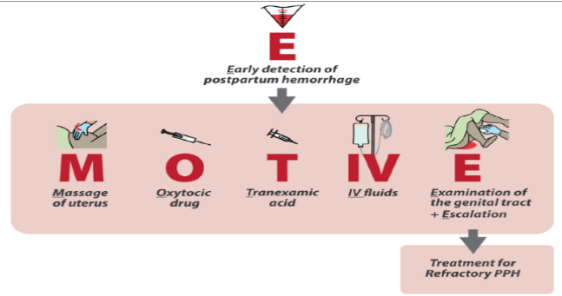


*We have already discussed how you detect PPH, here is an example of a calibrated drape.*

| **Show** picture of the calibrated drape to the participant (In the same way as described for the E-MOTIVE bundle diagram above) |
| --- |


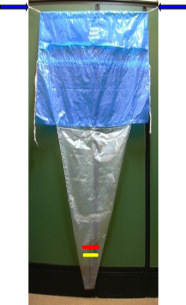


EMOTIVE drape

Yellow line 300ml

Red line 500ml

*This is a tool used to help measure blood loss to detect PPH. This drape is calibrated has visible line marking when 500 ml of blood has been lost.*

1. **How helpful do you think this would be for detecting of PPH by either yourself or team members?** *[Motivation/Beliefs about Consequences]*

*Prompt: Why or why not? How does this compare to how PPH is currently detected in this facility?*

1. **What would be needed to help introduce this calibrated drape in your facility?** *[Capability/Behaviour Regulation]*

Prompts: Would there be any challenges that you could anticipate?

*After PPH is detected, the treatments in the bundle are massage of uterus, use of oxytocics and tranexamic acid and giving IV fluids all delivered* ***all at once****.*

1. **Can I please confirm which of these you currently use in this facility?** *[Capability/Memory, Attention & Decision Processes]*
   1. **Do you use some treatments in the bundle more often than others? Why is that?**
2. **Does your team currently use any of these treatments all at once?**

*If yes, ask:*

- 1. **Did you and your team have to make any changes in order to do these treatments all at once? What are the challenges to using the treatments all at once?** *[Capability/Behaviour Regulation]*

*Prompts: What were these changes? Which treatments are used in combination or not?*

1. **How confident do you feel about performing all these treatments in the bundle at once?** *[Capability/Beliefs about Capabilities]*
2. **What would make you more or less confident?** *[Capability/Beliefs about Capabilities]*
3. **Are there any treatments you feel more or less confident doing?**
4. **In your opinion, are any of the treatments in the bundle more or less important than the others? Why is that?** *[Motivation/Goals]*
5. **Which of the treatments in the bundle are not currently used?** *[Capability/Memory, Attention & Decision Processes]*

*For* ***each*** *treatment not used (i.e. massage of uterus, use of oxytocins, use of tranexamic acid & giving IV fluids) mentioned ask:*

1. **Do you think there would be any challenges or issues to using [treatment]? What would those be?** *[Capability/Beliefs about Capabilities]*
2. **If you and your colleagues were asked to use these treatments all together when managing PPH, as described in the bundle, what would you need to help you do it?** *[Capability/ Beliefs about Capabilities]*
3. **To what extent would you know what to do?** *[Capability/Knowledge]*
4. **To what extent would you need additional skills?** *[Capabilities/Skills]*
5. **Would you have everything you need to perform the bundle?** *[Opportunity/Environmental Context & Resources]*
6. **What do you think are the potential benefits of managing PPH using such a bundle?** *[Motivation/Beliefs about Consequences]*
7. **What do you think are the potential disadvantages of managing PPH using such a bundle?** *[Motivation/Beliefs about Consequences]*
8. **To what extent do you think managing PPH as described in the bundle, is likely to help manage PPH more effectively?** *[Motivation/Beliefs about Consequences]*
9. **How would managing PPH as described in the bundle affect the ways you work as a team?** *[Motivation/Beliefs about Consequences]*

*Moving on the final part of the bundle*

1. **Does your team currently examine the woman’s genital tract as part of PPH management?** *[Capability/Memory, Attention & Decision Processes]*

*If yes, ask:*

1. **Who examines the woman’s genital tract?** *[Motivation/Professional Role & Identity]*
2. **How and when do you do the examination?** *[Capability/Memory, Attention & Decision Processes]*
3. **Are there any challenges to performing this examination? Does anything ever get in the way of you doing these examinations?** *[Capability/Beliefs about Capabilities]*

*If no, ask:*

1. **Why is it not done? Is there anything that makes it difficult to do?** *[Capability/Beliefs about Capabilities]*
2. **What would help you to do this in practice?** *[Capability/Beliefs about Capabilities]*

**Section 5. Conclusion**

*Thank you for your responses. We are now moving on to the final section of the interview where I have a few more general questions about PPH.*

1. **Does your team get any feedback on how you have detected and managed PPH?** *[Capability/Behaviour Regulation]*

*If yes, ask:*

1. **From whom?** *[Motivation/Professional Role & Identity]*
2. **What does the feedback cover?** *[Capability/Knowledge]*
3. **How useful do you feel this feedback is?** *[Motivation/Beliefs about Consequences]*
4. **What could be done to make this feedback more useful?** *[Motivation/Beliefs about Consequences]*

*If no, ask:*

1. **Do you think having feedback would be useful?** *[Motivation/Beliefs about Consequences]*
2. **How do you feel when you are managing a PPH? Why is that?** *[Motivation/Emotion]*

*Prompts: To what extent is managing PPH something you find stressful? Why is that?*

1. **How do you feel when something goes wrong or there is a complication?** *[Motivation/Emotion]*

*Prompt: Why is that? Is there anything that could be done to help or support you and your colleagues in this situation?*

1. **Have you ever been concerned about complaints from the community about the care they received?** *[Motivation/Emotion]*

*Prompts: What were the complaints? Why do you think they complained? How did you feel about the complaints if you were personally involved?*

1. **Do you know or have you ever heard of someone being disciplined for inadequate detection and/or management of PPH?** *[Motivation/Reinforcement]*
2. **To what extent does disciplinary action being taken on staff or on the hospital influence the detection and management of PPH in this facility?** *[Motivation/Reinforcement]*
3. **How does this impact on what you have told me about how staff do your job?**
4. **In your opinion, what do you think would happen if PPH was not detected and managed in your facility?** *[Motivation/Beliefs about Consequences]*

*Prompts: Would there be any consequences? For whom?*

1. **Are you aware of any strategies in your facility to try to improve detection and management of PPH?** *[Capability/Behaviour Regulation]*
2. **If yes, please explain and in your opinion, how effective have these strategies been?**
3. **Lastly, I would just like to quickly ask to what extent has the COVID-19 outbreak impacted on maternal care in your facility?**

*Prompts: Do fewer women come to the facility? Have maternity staff been seconded to other wards? Have you cared for women with Covid-19? Do you have to wear personal protection equipment? Have you received training on how to cope with Covid-19 in general maternity wards and for managing PPH?*

1. **Finally, is there anything else that you would like to share with me about anything we discussed today?**

| **Field notes:** Please remember to write down some brief field notes at the end of the interview. This provides helpful contextual information about the interview, that will help with analysis. Thank you. |
| --- |
| As you are conducting IDIs during a pandemic time, please, provide a bit of context:  On **procedures of contacting the respondents** (any challenges/ difficulties you faced? How did you overcome those? Did you have to contact the eligible respondent multiple times? Etc.) |
| **Interaction between interviewer and interviewee:** **How did you feel about the interactions between you and the respondent**? (i.e. was s/he spontaneous? Did you feel the respondent was shy/uncomfortable in providing detail on any Qs? Could you recognize any reasons of her/him being uncomfortable? Please, list all the possible reasons that you identified.  How did you manage those situations and encourage the respondent to respond? Do you think your professional background (being a health professional) had an influence on the respondent?) |
| **Researcher reflection and identification of bias:** Please, spend a few moments reflecting on the whole of the interview and your performance as a participant in the narrative and as an interviewer. **What went well/not well and why? Do you think you missed any important Qs/prompts?** If so, please note down immediately and make sure you ask those in your next interview. |

##

## Appendix 2

Table 1: Summary table of knowledge and current practices related to post-partum haemorrhage detection and management reported by the healthcare providers working in the maternity ward in Nigeria, Kenya, and South Africa (N=45). Verbatim quotations from participants are in quotations, and the author interpretations are in regular text. Table 4 in the main manuscript compares these reported current practices to the WHO recommended practices to elucidate where there are gaps between policy and practice.

| **Themes** | **Subthemes** | **Nigeria** | **Kenya** | **South Africa** | **Summary** |
| --- | --- | --- | --- | --- | --- |
| **Detection of PPH** | |  |  |  |  |
|  | **Factors for suspecting PPH** | **Visual estimation of blood loss (>500mLs) and changes in hemodynamic condition play vital roles in suspecting of PPH –** reported by the participants in Nigeria   - Based on the assessment of women’s medical history (anaemia and other risk factors) where available* (for women who booked early or accessed ANC services) - Observe excessive bleeding - For active/ excessive/ heavy/continuous (note: participants used these terms interchangeably) bleeding apply 4T (tone, tissue, trauma, and thrombosis) to identify cause of bleeding and suspect PPH - If uterus is not soft and contracted - After monitoring and assessing measurable signs including estimate >500mLs of blood loss after 30 minutes for next 2 hours - Any changes in the signs and symptoms of haemodynamic (fainting, pallor, shaky, dizzy) and any changes in the vital signs (BP and P) rate (PR) and other signs (pallor)s - Number of under pads soaked and changed. - For concealed PPH, woman will show fainting attack and look pale but no obvious blood loss. | **Active bleeding and changes in physical condition together play a vital role in suspecting of PPH –** reported by the participants in Kenya   - Based on the assessment of women’s previous and present medical history (prolonged labour, multiple pregnancies, any mother managed with syntocinon) and other clinical conditions, where available* (for women who booked early or accessed ANC services) - The most important factor to suspect PPH is excessive blood loss, which participants defined as active bleeding for ≥30 minutes following childbirth for first two hours (note: differs from the WHO PPH definition of blood loss >500mls during the first 24 hours post-partum). - For active bleeding, apply 4T (tone, tissue, trauma, and thrombosis concept) to identify cause of bleeding and suspect PPH - After monitoring and assessing measurable signs including estimate >500mLs of blood loss after 30 minutes for next 2 hours (note: this differs from the WHO definition of blood loss >500mlsduring the first 24 hours post-partum). - Rapid change in BP and PR and other vital signs - Presence of any remaining (like placental tissues and clots) following childbirth - Multiple and frequent changes of maternity pads - If the mother reports heavy bleeding | **Patient’s history, changes in physical condition and amount of blood loss play a vital role in suspecting of PPH –** reported by the participants in South Africa   - Based on the assessment of women’s previous and present medical history (previous history of PPH, genetic history, complicated delivery) where available* (for women who booked early or accessed ANC services) - Changes in the vital signs (changes in BP and pulse rate) and other physical signs and symptoms - Excessive blood loss following childbirth and rubbing up uterus cannot stop bleeding and the woman starts showing signs of shock/unconsciousness - Rapid/ multiple and frequent changes (note: participants used these terms interchangeably) of maternity pads | Visual estimation of blood loss and assessment of vital signs are the key methods applied to detect PPH in all three countries.  Doctors in Kenya and Nigeria mentioned applying 4Ts (tone, tissue, trauma, and thrombosis) to determine aetiology of PPH.  A few HCPs (doctors) in all three countries mentioned using a woman’s medical history to assess the risks for PPH. |
|  | **Steps for PPH detection mentioned by the participants** | - Following the childbirth and placenta being out, the healthcare providers do the following to detect PPH: - Monitored for active heavy vaginal bleeding; increased heart rate and patient will feel dizzy and cold - Examining if the placenta is completely out and no remaining is left - inspect genital tract if there is any laceration or tear - assess the uterus if it is contracted or flabby - Ensuring the bladder is empty - Monitor the woman’s vital signs - Assess the number of maternity pads/wrappers being used/changed. - Use kidney dishes, wrappers/pads to estimate/ measure blood loss (note: the terms ‘estimation of’ and ‘measuring’ blood loss were used interchangeably)   **Key facts:**   - Usually, a midwife/nurse suspects the PPH and is the first person to respond. - Sometimes doctors monitor and assess the woman and detect PPH - Other staff on duty will join in response to call for help. - Participants did not mention exact blood loss quantity. | - Collect patient’s history for risk factors and conduct physical examination - Midwife assisting the vaginal delivery will conduct general inspection after 3^rd^ stage of labour - Each woman would be given a maternity pad following childbirth - Assisting midwife/nurse should be proactive in monitoring the woman every 15-30 minutes for the first two hours: - the number of pads/ wrappers/ linens/gauzes has been used or changed. Advise the woman to inform the midwife if bleeding and midwife will re-examine. - Estimation/ measuring of blood loss using non-calibrated jugs, kidney dishes and maternity pads counts (note: the terms ‘estimation of’ and ‘measuring’ blood loss were used interchangeably) - Examine the vaginal canal for hematomas or tears. - any deviation in vitals- changes in BP and heart rate; if the uterus is contracted or not; the patient appears to be confused, if the patient been able to pass urine without catheter - Will make a judgement call if the woman loses approx. 200mLs of blood or if 4-5 pads were used within an hour after delivery or if the maternity pad soaked within 30 min to the next hour after delivery,   **Key facts**:   - In Kenya, midwives or nurses are responsible for first detecting PPH, but cannot fully diagnose PPH without a doctor’s confirmation. Midwives and nurses are the first responders, and doctors will join to confirm diagnosis and progress treatment | - Following childbirth, the woman needs to be observed and monitored - Check vital signs (BP and PR); - Examine the uterus to determine if it is contracted and the vagina for any tears or trauma lacerations. Then put a linen saver - Midwife would do “pad checks” to estimate “measure” blood loss following the local guideline/protocol used in the facility that includes (note: the terms ‘estimation of’ and ‘measuring’ blood loss were used interchangeably) - check the linen saver (a linen saver can soak about 300 to 400mL of blood) - count the number of pads changed (changing of 5 pads = 50-100 mLs of blood loss; called “mom check”). - check if blood pooling on floor underneath bed - Re-examine the uterus if it seems floppy if it is contracted find the source of bleeding - Re-check the vital signs if the patient starts showing signs of shock (tachycardia, dropping BP & high PR) - Call for help   **Key facts**:   - Usually, a midwife detects PPH and the first person to respond. Then the nurse joins in response to call for help. Then the doctor will be called - Pad check is conducted by midwives and nurses - doctors must confirm the PPH to proceed with treatment/ administer drugs | Midwives and nurses are the first responders of PPH detection in all three countries as they assist women with childbirth. However, in Nigeria sometimes doctors assist with childbirth as well and in tertiary hospitals in South Africa a doctor must be called to confirm PPH and proceed with treatment.  Detection of PPH depends on how closely the woman is monitored by the birth attendant(s). Estimated mls of blood loss per pad/ linen was mentioned by participants in Kenya and South Africa; no such information for Nigeria. |
|  | **Method and equipment used to detect PPH** | **Active bleeding; visual estimation of blood loss using**  **wrappers/ sanitary or maternity pads or under pads/ gauze/**  (Wrappers - 1-2 wrappers indicate mild, 3 wrappers indicate heavy bleeding or counting numbers of wrappers that soaks)  **Kidney dishes** or measuring jug  Observation of colour of blood  Waterproof delivery mat | **No standard measuring instruments used in the facility**  Visual estimation of blood loss  (One HCP improvised a normal saline IV container that was calibrated at 50- 500 mls to use as a PPH measuring tool; fill the blood inside the saline container to measure. Estimating blood loss visually is not standardized)  Linen, number of used pads and gauze  (If 4-5 pads were used within an hour after delivery, a judgement call would usually be made.)  Monitoring vital signs (such as check BP between 10-15 minutes after delivering the child and placenta  Measuring jar (Note: unclear if calibrated or non-calibrated) | **No standard measuring instruments used in the facility but Presence of protocols for the number of maternity pads used to detect PPH**  For visual estimation of blood loss:  Pad checks  +  Linen savers - (One linen saver can soak about 300-500mls blood)  +  check if blood pooling on floor underneath of bed.  +  Assess woman’s vital signs (BP; PR; shock; pale/ dizzy)  Use pack of swabs after delivery & suturing, returning after 15 minutes to check.  Some facilities do not have anything to measure blood loss | No standard PPH detection protocol was used by the HCPs in all three countries. Heavy vaginal bleeding, number of maternity pads or linen soaked and changes in vital signs were the major ‘tool’ in estimating blood loss that helped them detecting PPH. For concealed bleeding, HCPs relied on vital signs only. |
|  | **Challenges of visual estimation/ blood estimation** | - Subjective estimation of the amount of blood loss; hence, subject to inaccurate detection - Use of kidney dishes and wrappers or pads give inaccurate estimation of blood loss - Requires years of experience to become an expert in estimating of blood loss to suspect PPH - The process often delays the detection of PPH and can lead to fatal maternal deaths | - Requires experience to be an expert of using visual estimation to detect PPH using this method. Inexperienced clinical staff may not be able to recognise PPH and consider this as normal blood loss. - If the midwife does not assess the blood loss at the early stage, this may lead to under/overestimation of blood loss | - Subjective estimate of the amount of blood loss; hence, subject to provide inaccurate detection - Patients often throw pads and put a new pad on without informing the staff; hampers the total count of pads they used - Some midwives perceived no challenges | Participants mentioned that the key challenge to visual estimation of blood loss was its subjectivity. Visual estimation can be time consuming and gives inaccurate estimation of total amount of blood loss. Participants often used the terms ‘estimation’ and ‘measurement’ of blood loss interchangeably although they used mostly non-calibrated tools, limiting the reliability of ‘measurement’. |
|  | **Challenges or barriers to PPH detection** | - Unavailability of standard and objective method to quantify accurate blood loss. Reliance on subjective estimates of blood loss - Variation in the definition of volume of blood loss required to detect a PPH - Improving estimation based on blood estimated by counting number of maternity pads or collected in kidney dishes. - Measuring to estimate of blood loss varies person to person. Individuals have varying ways of measuring blood loss, e.g., weighing of soaked pads or use of calibrated containers. This can give over/under estimation of blood loss. - Estimation of blood loss requires visible blood loss. Sometimes the bleeding is concealed (e.g., uterine rupture during delivery). - Shortage of staff, unequal level of knowledge and skill sets and shortage of other essential resources - Shortage of staff interrupts early detection of PPH if there is a high number of deliveries on the ward at the same time. - Unequal level of expertise among staff, particularly among new and junior staff - Dealing with cases mis-managed by birth and maternity staff - Shortage of essential resources for PPH detection - Difficult to do the risk assessment for patients who do not come for ANC services or booked-in early - Detection of PPH can be challenging for unregistered or referred women with no medical history available. - PPH cannot be predicted by the ease of the labour and can happen suddenly. Hence, medical history is important - Medical history of the women is important as woman may not show any early symptoms for PPH*.* - Late referrals of women from lower-level facilities or community without complete patient history and information about the volume of blood loss before check-in into the higher-level facility - Lack of knowledge about PPH among pregnant women. They often do not realise they are bleeding and do not inform HCPs immediately.   **Alternative opinions:**   - Detecting PPH is not challenging - The challenge is not detecting PPH but with managing it. | - Unavailability of standard and objective method to quantify accurate blood loss. Reliance on subjective estimate of blood loss - Lack of accurate measurement method for blood loss. - Detection is easy when a PPH occurs in the delivery room. It is harder if the blood loss was 200mls after delivery and later things suddenly change. - Relying on blood loss estimation is not always effective. Some women experience PPH without losing recommended amount of blood loss (around 250 mLs) but show dramatic changes in their vitals*.* - Shortage of staff, unequal level of knowledge and skill sets and shortage of other essential resources - Not having clear understanding about PPH among HCPs can be a challenge. Particularly, not all 1^st^ responders to PPH have sufficient knowledge and expertise. - If the 1^st^ responders (i.e., the midwife) does not observe and assess the blood loss and monitor vital signs properly after the delivery. - Shortage of trained and qualified staff in the maternity ward, particularly when there are multiple women in different stages of labour with different obstetric emergencies. - Difficult to do the risk assessment for patients who do not come for ANC services or booked-in early - Detection of PPH can be challenging for unregistered women with no medical history available. - Women usually do not access facility delivery services. Unbooked or late referrals of women from lower-level facilities or community often do not have information about the level of blood loss. - Unavailability of doctors’ delays detecting PPH as they make the final call (i.e. confirm PPH) - Lack of knowledge about PPH among pregnant women. They often do not realise they are bleeding and do not inform HCPs immediately. Sometime women do not cooperate with nurses/midwifes for PPH detection.   **Alternative opinions:**   - No challenges for booked women because all staff are trained to monitor vitals and observe BP going down. | - Unavailability of standard and objective method to quantify accurate blood loss. Reliance on subjective measurement of blood loss. - Estimation of blood loss varies person to person, hence subjective and not always accurate - Pad check is the most commonly used method that is not easy to apply. Patients often throw pads and put a new pad on without informing the staff and this hampers the total count of pads they used. - For pad check, urine output on the pad needs to be monitored which is difficult - Estimation of blood loss requires visible blood loss. Detecting a PPH is challenging when there is no visible active blood loss. - Shortage of staff, unequal level of knowledge and skill sets and shortage of other essential resources - Shortage of staff: Due to lack of staff, often staff need to move one mother to another. By the time they return to the previous mother, sometimes they missed the opportunity for early detection and find the mother at a later stage of PPH as attending a delivery patient takes time. - Differences in understanding in the importance of the blood flow; people react less to a low or no visible blood loss. - Difficult to do the risk assessment for patients who do not come for ANC services or booked-in early - Having no patient history for pregnant women with high-risk factors is a challenge for early detection of PPH - PPH cannot be predicted by the ease of the labour and can happen suddenly - Unavailability of doctors’ delays detecting PPH as they make the final call (i.e. confirm PPH). - At district hospital, doctors do not attend pregnant women and often be contacted via phone and they confirm PPH cases over the phone. - For a doctor, who has not delivered and not knowing how much blood has been lost, it can be challenging to determine PPH - Not all staff (particularly new staff) have similar level of knowledge and expertise. Shortage of trained and qualified staff in the maternity ward, particularly when there are multiple women in different stages of labour - Lack of knowledge about PPH among pregnant women. They often do not realise they are bleeding and do not inform HCPs immediately. - Poor or lack of communication among staff within facility or between referral and referred facility staff or between staff and patient is another challenge of PPH detection - Staff at the lower-level facility often underestimate the amount of blood loss following childbirth and often fail to detect PPH timely and refer the patient immediately.   **Alternative opinions:**   - Detecting PPH is not challenging- *“*you just know*”* | Absence of standard and objective method to quantify blood loss for PPH in all three countries is the key challenge for PPH detection. Detection can be delayed because of reliance on only vital signs to identify a PPH.  Detection of PPH becomes more challenging for women who do not have medical history, booked late, and late referral without complete patient information. However, a few participants perceived PPH detection was not challenging as their staff were skilled. |
| **Management of PPH** | | | | | |
|  | Use of protocol or Guidelines | Use of national or international (RCOG) guidelines for PPH prevention and management; but no standard guideline for PPH management.  Local guidelines are not up to date with recent evidence  Guidelines are not always context-specific (country and health facility specific); hence, not always easily implementable.  Either online or hard-copy version of protocols/guidelines are available. No common way to share guideline with all HCPs (shared by a resident doctor who downloaded and shared it via WhatsApp)  Some 1st responders were unaware of the existence of protocol(s)  Pictorial display (posters) of PPH management at labour room/ward  HCPs mainly doctors revisit protocols if there is any doubt about any procedures. Visiting protocols is important for new/junior staff | Presence of national and international (WHO guideline) guidelines and SOPs for PPH prevention and management. Also, BEmONC* booklets are available in some facilities. However, BEmONC guidelines are more known to the HCPs  Not all facilities have reviewed/ updated local guidelines  Protocols/guidelines are mostly available as hard-copy or poster version  Presence of SOPs for PPH management in the Labour ward which are perceived useful  Pictorial display (posters) of PPH management at the maternity or labour room/ward to ensure easy access of the staff | Presence of national (2016) and international (WHO guideline) guidelines and SOPs for PPH prevention and management at tertiary level hospitals. At District hospitals, guidelines are reported not easily accessible and not used frequently.  ESMOE algorithms for PPH management is commonly used  Not all hospitals including tertiary level hospitals have reviewed/ updated guidelines  Doctors are more likely to be aware of guidelines compared to the first responders to PPH  First responders to PPH are aware of posters (displayed at labour ward) and EmOC material  Tertiary and District hospitals usually have pictorial display (posters) of PPH management at the maternity or labour room/ward and near nurse station to ensure easy access of the staff. For secondary facilities, posters may not be available.  Guidelines are used irregularly. These are for young doctors/ staff because senior staff know what to do so work on autopilot. | All tertiary level facilities use  international guidelines for PPH management but claimed guidelines may not to be context (place/ country) specific. Lack of specificity hinders implementation of guidelines. Not all facilities adapt the guideline to the local context.  Existing local and national guidelines need to be updated with recent evidence.  Pictorial display of PPH management steps at the workstation were seemed to ensure easy accessibility of guidelines for junior doctors, midwives, and nurses. However, they do not revisit the guideline regularly. |
|  | Steps of PPH management | **After suspecting PPH**   - Called for help including doctor as it requires a teamwork - Same time secure 2-line wide bore canula to administer uterotonic (oxytocin/ misoprostol) and IV fluids - Continue uterus massage to ensure it has contracted; also try to evacuate the uterus and empty the bladder - Monitor woman’s vital signs including BP and PR - Administer TXA (subject to availability/ purchase-ability of woman’s family) - Examine genital track for any laceration or tear - Collect blood sample for PCV, grouping and matching - Empty the bladder - Put anti-shock garment if the woman goes into shock   **Common drugs and PPH related equipment used:**   - IV fluids - Oxytocics (Oxytocin; misoprostol; ergometrine - Anti-shock garments - Intrauterine balloon tamponade   If bleeding continues, the women may go into shock.   - The patient should be kept hydrated and warm - Apply anti-shock garment - Need urgent PCV and transfuse blood - Give oxygen   **Key fact**:   - No calibrated tool is used for measuring blood loss - Uterine massage, IV fluids and oxytocics are routinely used as first line treatment - TXA was used in rare occasion if oxytocics failed to stop bleeding. - Mostly women’s relatives purchased TXA from outside the facility - Genital tract examination is routinely applied to detect the cause of bleeding and usually a doctor performs this examination - Not all midwives have enough skill performing genital tract examination - Some women refuse to have an examination of the genital tract due to associated pain - NASG used to resuscitate a woman with PPH; - Advanced teamwork environment present to manage PPH. - Nurses and midwives were inconsistent regarding the amount of dosage for 1^st^ line drugs should be administered. | **After suspecting PPH**   - Call for help as it requires teamwork - Rub uterus and checked for retain birth products - Remove clots and put catheter to drain the urine - Call the duty doctor to lead and to manage any difficulties - Secure 2-line wide bore canula to administer uterotonic (oxytocin/ misoprostol/ syntocinon) and IV fluids Check for vital signs (BP and PR) - Collect blood sample for grouping and matching and take preparation for blood transfusion - Apply UBT (if not available use male catheter) - Keep the mother warm   **Common drugs and PPH related equipment used:**   - IV fluids - Oxytocics (Oxytocin; misoprostol if oxytocin does not work; syntocinon) - TXA - Anti-shock garments - Intrauterine balloon tamponade   **Key facts**:   - No calibrated tool is used for measuring blood loss - Uterine massage and oxytocics are routinely used as first line treatment and are used simultaneously - IV fluids are routinely used either as a first line of treatment for managing PPH or after massaging the uterus - TXA is not routinely used and not used as first-line drug. - Genital tract examination is routinely applied to detect the cause of bleeding and usually a doctor performs this examination - Not all midwives have enough skill performing genital tract examination - Some women refuse to have an examination of the genital tract due to associated pain - UBT is used however, essential equipment for UBT is not available in every facility. - Nurse or midwife cannot do tear repairing | **After suspecting PPH**   - Midwife, who is attending the woman, would remove the placenta and the membranes - Checked for uterus if it was contracted and apply uterine massage - Called for help (because you cannot work alone) including other midwifery staff and doctor - Once the help arrives, put up 2 big IV lines and administer oxytocics – one line to administer 20-unit IV fluids and another one to give 10 units of oxytocin to manage PPH and oxytocics (oxytocin or misoprostol)/ cyclokapron (TXA) - Started rubbing the uterus making sure that it stays contracted - checked and monitored the vital signs - another staff checked the vagina of the patient again for any retained products. She found out a piece of retained placenta and tried the manual removal of the membrane. - if bleeding continues, check for trauma lacerations and tears to suture - ask the doctor if Cyklocapron (TXA) is needed - ask for D & C pack (kept in theatre) - Then doctor would be called. - Ask someone to draw bloods, to ensure the blood group - The woman will be taken into the theatre for cervical tear upon confirmation from the doctor - In case the doctor is busy in attending other emergency patients (may be in the theatre), the attending staff (nurse/ midwife) would call the doctor and depending on the volume of how much blood they say over the phone, the doctor would instruct them to give an oxytocin infusion to help with uterine contraction - The doctor inspected conjunctival pallor, took the blood pressure, pulse and requested urgent ward Hb and take blood as well for a formal Hb while awaiting. - The doctor performed a quick vaginal examination to see the source of bleeding (the blood might be coming from the cervix or inside the vagina due to lacerations or wound);   **Common drugs and PPH related equipment used:**   - IV fluids - Oxytocin/misoprostol/ syntocinon - TXA (Cyclokapron) - Uterine balloon tamponade   **Key facts:**   - Facility staff follow ESMOE algorithm for PPH management - Usually, a midwife detects PPH and the first person to respond. Then the nurse joins in response to call for help. The doctor will be called last. - At District hospitals midwives and nurses could perform the first-response and an advanced midwife can even order and administer TXA; whereas at the tertiary level hospital a doctor will be engaged in the first-response and administering the drugs. - Have team leader to make sure all steps are followed as per protocol - No calibrated tool is used for measuring blood loss - Uterine massage and setting up IV lines to administer drugs used as first-line treatment. Drugs will be administered upon doctors’ prescription - IV fluids are routinely used either as a first line of treatment for managing PPH or after massaging the uterus - TXA is not routinely used as first-line drug - Genital tract examination is currently done to detect cause of bleeding - Not all midwives or nurses have enough skill performing genital tract examination - Some women, particularly young mothers, refuse to have a genital examination - Genital tract examination can be challenging due to inadequate light in the labour ward and the beds are low, the staff has to bend. | Overall, in all three countries there was congruence of clinical management with published PPH Guidelines. Midwives, nurses and doctors are usually involved in the first response of primary PPH management. However, at District Hospitals in South Africa, midwifery staff were found to be more actively involved in the first response of PPH treatment including TXA administration.  Oxytocic (oxytocin, misoprostol) is commonly used but TXA is more commonly used in Kenya and South Africa but not as first-line drug. |
|  | Challenges/ barriers of PPH management | **Within facility:**   - Unequal or mixed clinical understanding on PPH management - Subjective method of PPH detection delays PPH management - Lack of resources within facility for PPH management - Shortage of staff - Unequal level of knowledge and expertise among staff (new/ junior staff) - Shortage of essential drugs and equipment - Challenges of maintaining cold chain for oxytocins - Inadequate blood supply within facility - Delay in procuring essential drugs and blood outside the facility - High cost of TXA and poor-quality brands for oxytocins - PPH management guidelines are not up-to-date and context-specific - Inadequate capacity of the tertiary level facility (to accommodate high-volume of critical patients)   **Outside facility level:**   - Late referral of patients from the lower-level facilities or community - Incomplete patient history for unbooked or referred pregnant women   **Women level challenges**   - Non-cooperation from women during implementing clinical interventions (genital tract exam) - Delay in seeking consent from women or her relatives - Quick and sudden onset of PPH without visible risk factors | **Within facility:**   - Unequal or mixed clinical understanding on PPH management - Subjective method of PPH detection affect PPH management - Lack of resources for PPH management - Shortage of staff - Unequal level of knowledge and expertise among staff (new/ junior staff) - Shortage of essential drugs and equipment (no stirrup bed, low lighting system to examine the woman). - Inadequate blood supply within facility - Delay in procuring essential drugs and blood outside the facility - PPH management guidelines are not up-to-date and context-specific - Inadequate capacity of the tertiary level facility (to accommodate high-volume of critical patients) - Absence of improvement strategies within facilities targeting PPH management   **Outside facility level**   - Late referral of patients from the lower-level facilities or community - Incomplete patient history for unbooked or referred pregnant women   **Women level challenges**   - Non-cooperation from women during implementing clinical interventions (genital tract exam) - Delay in seeking consent from women or her relatives - Quick and sudden onset of PPH without visible risk factors | **Within facility:**   - Unequal or mixed clinical understanding on PPH management - Subjective method of PPH detection affect PPH management - Lack of resources for PPH management - Shortage of staff - Unequal level of knowledge and expertise among staff (new/ junior staff) - Shortage of essential drugs and equipment (e.g UBT) - Inadequate blood supply within facility - Delay in procuring essential drugs and blood outside the facility - Challenges of maintaining cold chain for oxytocins - PPH management guidelines are not up-to-date and context-specific - Inadequate capacity of the tertiary level facility (to accommodate high-volume of critical patients) - Absence of improvement strategies within facilities targeting PPH management   **Outside facility level:**   - Late referral of patients from the lower-level facilities or community - Incomplete patient history for unbooked or referred pregnant women - Miscommunication between lower and higher-level facilities   **Women level challenges**   - Non-cooperation from women during implementing clinical interventions (genital exam) - Quick and sudden onset of PPH without visible risk factors | Challenges or barriers of PPH management seemed to be common across three countries, this includes lack or shortage of essential resources (staff; killed staff; consumables; drugs; blood supply; capacity of accommodating multiple emergencies; late referral etc). |

*For women who booked early or who accessed ANC services, medical history is available. For unbooked and referred cases, the facility may not have patients’ history.; BEmONC: Basic Emergency Obstetric and Newborn Care; BP: Blood pressure; ESMOE: Essential Steps in the Management of Obstetric Emergencies; HCP= Healthcare providers; PR: Pulse rate; PCV: Packed cell volume; RCOG= Royal College of Obstetricians and Gynaecologists; TXA: Tranexamic acid; WHO – World Health Organization; SOP- Standard operating procedure.
